# Reciprocal regulation of LINC00941 and SOX2 promotes progression of esophageal squamous cell carcinoma

**DOI:** 10.1038/s41419-023-05605-6

**Published:** 2023-01-30

**Authors:** Jun-Tao Lu, Zhao-Yang Yan, Tong-Xin Xu, Fan Zhao, Lei Liu, Fei Li, Wei Guo

**Affiliations:** 1 Laboratory of Pathology, Hebei Cancer Institute, The Fourth Hospital of Hebei Medical University, Shijiazhuang, Hebei, China; 2 Department of Thoracic Surgery, The Fourth Hospital of Hebei Medical University, Shijiazhuang, Hebei, China; 3 Department of CT&MRI, The Fourth Hospital of Hebei Medical University, Shijiazhuang, Hebei, China

**Keywords:** Oncogenes, Tumour biomarkers, Targeted therapies, Long non-coding RNAs

## Abstract

LINC00941 is a novel long noncoding RNA (lncRNA) and emerging as an important factor in cancer development. However, the exact function and relative regulatory mechanism of LINC00941 in carcinogenesis of esophageal squamous cell carcinoma (ESCC) remain to be further clarified. The present study was to investigate the expression level, functions, and mechanisms of LINC00941 in ESCC tumorigenesis. LINC00941 was significantly upregulated in ESCC, and upregulated LINC00941 was correlated with dismal patient outcomes. LINC00941 functioned as an oncogene by promoting cells proliferation, stemness, migration, and invasion in ESCC. In terms of mechanisms, SOX2 could bind directly to the promoter region of *LINC00941* and activate its transcription. In turn, LINC00941 upregulated SOX2 through interacting with interleukin enhancer binding factor 2 (ILF2) and Y-box binding protein 1 (YBX1) at the transcriptional and post-transcriptional levels. LINC00941 recruited ILF2 and YBX1 to the promoter region of *SOX2*, leading to upregulation of the transcription of *SOX2*. Moreover, LINC00941 could promote the binding ability of ILF2 and YBX1 on mRNA of *SOX2* and further stabilize *SOX2* mRNA. Therefore, LINC00941 contributed to the malignant behaviors of ESCC cells via the unrestricted increase in SOX2 expression. In conclusion, our data indicate that LINC00941 exacerbates ESCC progression through forming a LINC00941-ILF2/YBX1-SOX2 positive feedback loop, and LINC00941 may be a promising prognostic and therapeutic target for ESCC.

## Introduction

Esophageal cancer is a heterogeneous disease with different biological, molecular and histological characteristics. Based on histological classification, esophageal cancer can be divided into esophageal adenocarcinoma (EAC) and esophageal squamous cell carcinoma (ESCC) [1]. ESCC accounts for over 90% of esophageal cancer cases in Asia, especially in China [1, 2]. ESCC is an aggressive subtype and is characterized by its progressive clinical course and high mortality. Despite tremendous advances in diagnostic and therapeutic approaches, the 5-year survival rate of ESCC patients remains dismal in recent decades [3, 4]. It is essential to explore the underlying molecular mechanisms in ESCC progression, which may be helpful for the development of effective therapeutic strategies to improve the prognosis of ESCC patients.

Genome-wide cancer analyses and functional studies are revealing an extensive landscape of functional mechanisms within the long noncoding RNAs (lncRNAs), which are defined as RNAs longer than 200 nucleotides that cannot be translated into proteins but can produce noncoding transcripts [5]. LncRNAs are important regulators driving many cancer phenotypes, such as proliferation, plasticity, invasion, and migration [6]. Mechanistically, the regulating effect of lncRNAs on gene expression and protein functions mainly depend on their localization and their interactions with DNA, RNA, and proteins [7].

LINC00941 is a novel lncRNA located on chromosome 12p11.21, with no coding potentiality [8, 9]. LINC00941 was recently identified as an oncogenic driver in multiple cancers [10–12]. For example, LINC00941 activates Hippo pathway through interacting with MST1 and subsequently enhances glycolysis in pancreatic cancer [10]. LINC00941 forms a complex with SMAD4 and prevents degradation of SMAD4 protein, resulting in activation of TGF-β/SMAD2/3 signaling pathway and consequently promotes metastasis of colorectal cancer [11]. LINC00941 also functions as an oncogenic gene in ESCC via sponging miR-877-3p in the cytoplasm to facilitate PMEPA1 expression [12]. However, where or how LINC00941 plays a pivotal role in controlling ESCC progression has not been fully elucidated. In this study, we characterized the functional roles and unexpected molecular mechanisms of LINC00941 in ESCC progression.

## Materials and methods

### Patients and specimens

Primary ESCC tissues and adjacent corresponding normal tissues were obtained from 80 ESCC patients who underwent surgical treatment at the Fourth Hospital of Hebei Medical University from 2012 to 2016. All written informed consents were obtained from all patients and the patients had not received any radiotherapy, chemotherapy, or biotherapy before operation. The surgical resection tissues were divided into two parallel parts: one part was fixed in formalin and embedded in paraffin; the other part was frozen and preserved at −80 °C. Clinicopathological features and clinical data were obtained from pathological diagnosis and hospital records (Supplementary table 1). The study was approved by the Ethics Committee of the Fourth Hospital of Hebei Medical University.

### Cell culture and treatment

The human ESCC cell lines KYSE-170, KYSE-150, TE-1, and YES-2 were obtained from China Center for Type Culture Collection (CCTCC, Wuhan, China). All cell lines were authenticated by short tandem repeat profiling and tested for mycoplasma contamination. Cells were grown in RPMI 1640 (Invitrogen, Carlsbad, CA, USA) supplemented with 10% fetal bovine serum (FBS, Invitrogen) and cultured in 5% CO_2_ at 37 °C. For blocking transcription, cells were treated with 5 µg/mL actinomycin D (Sigma, St. Louis, MO, USA) for 2, 4, or 6 h.

### Lentiviral infection

Human full-length LINC00941 (NR_040245.1) was amplified by reverse transcription-polymerase chain reaction (RT-PCR) and subsequently cloned into the lentiviral vector pCDH-CMV-MCS-EF1-Puro (pCDH). Then the lentiviral vector was co-transfected with two packaging vectors into HEK293T cells with Lipofectamine 2000 (Invitrogen). After transfection for 48 h, the lentivirus supernatants were collected. TE-1 cells were infected with the resultant viral supernatant for 72 h. These cells were selected in 1.0 μg/mL puromycin for 2 weeks to establish stable LINC00941-overexpressing TE1 cells.

### Plasmids and small interfering RNA transfection

The human full-length cDNA of *LINC00941*, *SOX2*, *ILF2*, and *YBX1* were obtained by RT-PCR and subcloned into the pcDNA3.1 vector. The small interfering RNAs (siRNAs) targeting human *SOX2* (siSOX2#1 and siSOX2#2), *ILF2* (siILF2), *YBX1* (siYBX1), and negative control siRNA (siControl) were purchased from Genepharma (Shanghai, China). The siRNAs targeting human LINC00941 (siLINC00941#1 and siLINC00941#2) were purchased from RiboBio (Guangzhou, China). Lipofectamine 2000 (Invitrogen) was used for transfecting plasmids and siRNAs into cells according to the manufacturer’s specifications. The sequences of siRNAs were listed in Supplementary Table 2.

### RNA extraction and quantitative RT-PCR assays

Total RNA extraction, reverse transcription (RT), quantitative PCR (qPCR), and quantification of target gene expression were performed as described previously [13]. The GoTaq®qPCRMaster Mix (Promega, Madison, WI, USA) was used for qPCR in accordance with the manufacturer’s protocol. GAPDH was utilized as an internal control to normalize gene expression. The sequences of primers were listed in Supplementary Table 3.

### Cell proliferation and colony formation assays

The cell proliferation ability was examined using MTS and colony formation assays. For the MTS assay, cells were inoculated in 96-well plates at a density of 1000 cells/well and grown for 0, 24, 48, 72, and 96 h. Then, the cells were incubated with 20 μl MTS solution (500 μg/ml) for 2 h, and the absorbance was measured at 490 nm. For the colony-formation assay, cells were inoculated in six-well plates at a density of 1000 cells/well and grown until cell clusters were visible. Then, the cell colonies were treated with 4% paraformaldehyde and stained with 0.1% crystal violet. Colonies composed of at least 50 cells were counted under microscope.

### Tumor sphere formation assay

Cells were inoculated in ultra-low attachment six-well plates at a density of 5000 cells/well, and cultured in DMEM/F12 (Invitrogen) supplemented with 2% B27 (Invitrogen), 20 ng/ml EGF (Invitrogen), 20 ng/ml basic FGF (Invitrogen), and 4 μg/mL insulin (Sigma) for 14 days. The spheres were photographed and counted under microscope.

### Cell migration and invasion assays

Cell migration ability was measured by wound healing assay and transwell chambers assay. For wound healing assay, cells were grown to 80% confluence in six-well plates and starved with serum-free medium. Then, the monolayers of cells were scratched by a pipette and cultured in medium containing 1% FBS for 24 h. The distance migrated by the cells into the wound was measured. The Matrigel-uncoated Transwell inserts or Matrigel-coated Transwell inserts (BD Biosciences, San Jose, CA) were used to measure the migration or invasion ability of cells as described previously (14). The number of migrating or invading cells was counted in five predetermined fields for each membrane at ×400 magnification.

### Chromatin immunoprecipitation-qPCR

Chromatin immunoprecipitation (ChIP)-qPCR assays were performed as previously described [14]. The promoter regions of *LINC00941* containing or lacking putative SOX2-binding element were enriched using an anti-SOX2 antibody in cells transfected with plasmids expressing SOX2. The promoter region of *SOX2* containing an YBX1-binding element was enriched using an anti-YBX1 antibody in cells transfected with plasmids expressing YBX1. The primers for ChIP-qPCR were listed in Supplementary Table 4.

### Dual-luciferase reporter assay

Using genomic DNA extracted from KYSE170 cells as DNA templates, the promoter regions of *LINC00941* containing or lacking a SOX2-binding element were amplified by PCR and subsequently inserted into the pGL3-Basic vector (Promega), and the promoter regions of *SOX2* containing or lacking an YBX1-binding element were inserted into the pGL3-Basic vector. Synthesis of the point mutation of SOX2-binding site on *LINC00941* promoter was performed using a Q5^®^ Site-Directed Mutagenesis Kit (New England Biolabs, Beijing, China). All constructs were verified by sequencing. The luciferase reporter constructs were co-transfected with pRL-TK plasmid into ESCC cells. The luciferase activity was measured using the Dual-Luciferase Reporter Assay System (Promega) and normalized using Renilla luciferase activity.

### Immunoblot and immunofluorescence assays

Immunoblot and immunofluorescence assays were performed as previously described [14]. The primary and secondary antibodies for immunoblot and immunofluorescence were described in Supplementary Table 5. Full and uncropped immunoblots were presented in Supplemental File.

### Subcellular RNA fractionation

Nuclear and cytoplasmic RNA was isolated and purified according to the manufacturer’s protocol of the PARISTM Kit Protein and RNA Isolation System (Invitrogen). The expression of LINC00941 in different subcellular fractionations was detected by quantitative RT-PCR (qRT-PCR) method.

### Fluorescence in situ hybridization

Staining of LINC00941 in cultured cells or paraffin-embedded tissues were performed according to the manufacturer’s protocol of the Ribo^TM^ Fluorescent in Situ Hybridization Kit (RiboBio). Fluorescence-conjugated LINC00941 Fluorescence in situ hybridization (FISH) probes were designed and synthesized by RiboBio. Briefly, cells were fixed in 4% paraformaldehyde for 10 min. After washing with PBS, the cells were permeabilized in 0.5% Triton X-100 for 5 min. The cells were then incubated with hybridization solution containing the LINC00941 probe overnight at 42 °C. Subsequently, the cells were washed separately with 4× SSC, 2× SSC, and 1× SSC. After the hybridization, the cells were counterstained with 4′, 6-diamidino-2-phenylindole (DAPI). Paraffin-embedded sections were deparaffinized and rehydrated. After washing with PBS, the tissues were permeabilized using protease for 20 min at room temperature. Next, the tissues were further incubated with hybridization solution containing the LINC00941 probe overnight at 42 °C. The next day, the tissues were washed with SSC and counterstained DAPI. Images were obtained with Olympus FV3000 confocal microscope.

### RNA pull-down and mass spectrometry analysis

The RNA pull-down assay was performed according to the manufacturer’s protocol of the Pierce™ Magnetic RNA-Protein Pull-Down Kit (Thermo). Briefly, the sense and antisense sequence of LINC00941 were transcribed in vitro using the T7 RiboMAX™ Express Large Scale RNA Production System (Promega), and biotinylated using Pierce RNA 3′ End Desthiobiotinylation Kit (Thermo). Subsequently, proteins extract from ESCC cells were incubated with biotinylated RNA, followed by incubation with streptavidin beads. Total proteins against LINC00941 sense or antisense strand were subjected to MS or Western blot analysis. Mass spectrometry (MS) was performed by Applied Protein Technology (Shanghai, China). The LINC00941-bound proteins with top 50 matching scores identified by mass spectrometry were listed in Supplementary Table 6.

### RNA-protein immunoprecipitation analysis

The RNA-protein immunoprecipitation (RIP) assay was performed according to the manufacturer’s instructions of the Magna RIP™ RNA-Binding Protein Immunoprecipitation Kit (Millipore, Billerica, MA, USA). In brief, ESCC cells were transfected with the plasmids expressing ILF2 or YBX1, and then lysed by RIP lysis buffer. The cell lysates were incubated with magnetic beads which already conjugated with anti-ILF2, anti-YBX1, or IgG overnight at 4 °C. After digestion with protease K, the immunoprecipitated RNA was subjected to qRT-PCR analysis.

### Protein immunoprecipitation

KYSE170 cells, transfected with the plasmids expressing ILF2, were lysed by ice-cold NP40 lysis buffer. After concentration, the cell lysates were incubated with anti-ILF2 antibody overnight at 4 °C to form the immunoprecipitation complex, and then incubated with pre-treated protein A/G beads for 1 h at room temperature. Normal rabbit IgG was used as a negative control. After washed three times with NP40 buffer, the immunoprecipitation complexes were eluted and boiled with sample-loading buffer for immunoblot detection.

### Animal experiments

To establish the subcutaneous xenograft tumor model, a total of 5 × 10^6^ LINC00941-overexpressing TE1 cells or control cells were injected into the dorsal flanks of 6-week-old male BALB/c-nude mice (*n* = 5 per group). The mice were killed after 30 days, and terminal volume and weight of tumor tissues were measured. Tumor volume was calculated with Volume = 0.5 × width^2^ × length. To establish the lung metastatic model, a total of 2.5 × 10^5^ LINC00941-overexpressing TE1 cells or control cells were intravenously injected into 6-week-old male BALB/c-nude mice via the tail vein (*n* = 5 per group). The mice were killed at day 30 after injection. The number of lung nodules was determined by hematoxylin and eosin (H&E) of paraffin-embedded tissue sections. Animals of similar age and weight were randomly selected and grouped. Histological evaluation was performed in a blinded fashion. The protocols for animal experimentation described in this paper were approved by the Animal Committee of the Fourth Hospital of Hebei Medical University.

### Statistical analysis

All in vitro experiments were performed three independent experiments, and data were presented as the mean ± standard deviation (SD). Two-tailed Student’s *t* test or the rank sum test was used to compare the differences between the experimental and control groups. Clinicopathologic significance in clinical samples was evaluated by Chi-square test for categorical data. Survival analysis was evaluated using Kaplan-Meier survival curves, and the log-rank test was used to assess statistical significance. Differences with *P* < 0.05 were considered statistically significant.

## Results

### Upregulation of LINC00941 in ESCC tissues and esophageal cancer cells

**Fig. 1:**
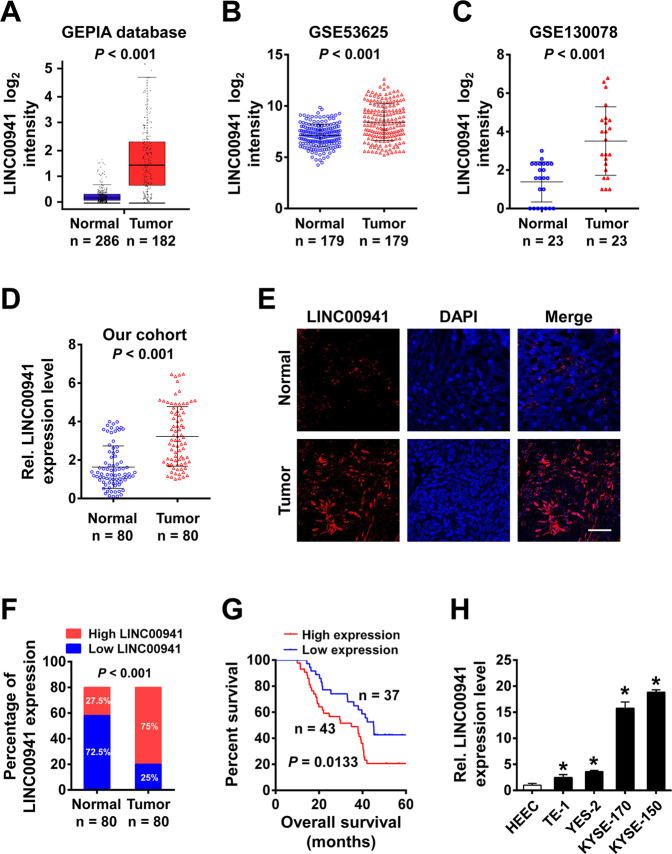
Upregulated LINC00941 predicts poor prognosis of ESCC patients. **A** Expression levels of LINC00941 in 182 esophageal carcinoma (ESCA) samples and 286 normal controls from GEPIA cohort. **B**–**D** The elevation of LINC00941 was validated by additional three ESCC cohorts, including GSE53625 cohort (**B**, 179 normal tissues vs. 179 tumor tissues), GSE130078 cohort (**C**, 23 normal tissues vs. 23 tumor tissues), and our in-house cohort (**D**, 80 pairs of ESCC tissues and adjacent normal tissues). **E**, **F** Representative images (**E**) and statistical analysis (**F**) of RNA FISH staining of LINC00941 in 80 paired ESCC tissues and adjacent normal tissues. Nuclei are stained with DAPI. Scale bars, 50 μm. **G** Kaplan–Meier curves of LINC00941 in ESCC patients for overall survival (OS). **H** Expression levels of LINC00941 in normal esophageal epithelial cell line and ESCC cell lines were determined by qRT-PCR. HEEC: Human normal esophageal epithelial cell. *P < 0.05.

The expression level of LINC00941 was significantly increased in esophageal carcinoma tissues compared to normal controls in GEPIA dataset (including 182 ESCA tumor samples and 286 normal controls; *P* < 0.05; Fig. 1A). The similar expression tendency of LINC00941 was also verified in GSE53625 and GSE130078 datasets (Fig. 1B, C). Upregulation of LINC00941 in ESCC tissues was also detected by RT-qPCR and FISH in the present study (*P* < 0.05; Fig. 1D–F). We further divided the patients into high (above the median, *n* = 43) and low (below the median, *n* = 37) LINC00941 expression groups according to the median value of LINC00941 expression levels detected by RT-qPCR. ESCC patients with high expression level of LINC00941 demonstrated poor five-year survival rate compared to patients with low expression level of LINC00941 (*P* < 0.05; Fig. 1G). The expression level of LINC00941 in ESCC cell lines was also higher than that in normal esophageal epithelial cells (Fig. 1H).

### LINC00941 promotes esophageal cancer cells proliferation, stemness, migration, and invasion

**Fig. 2:**
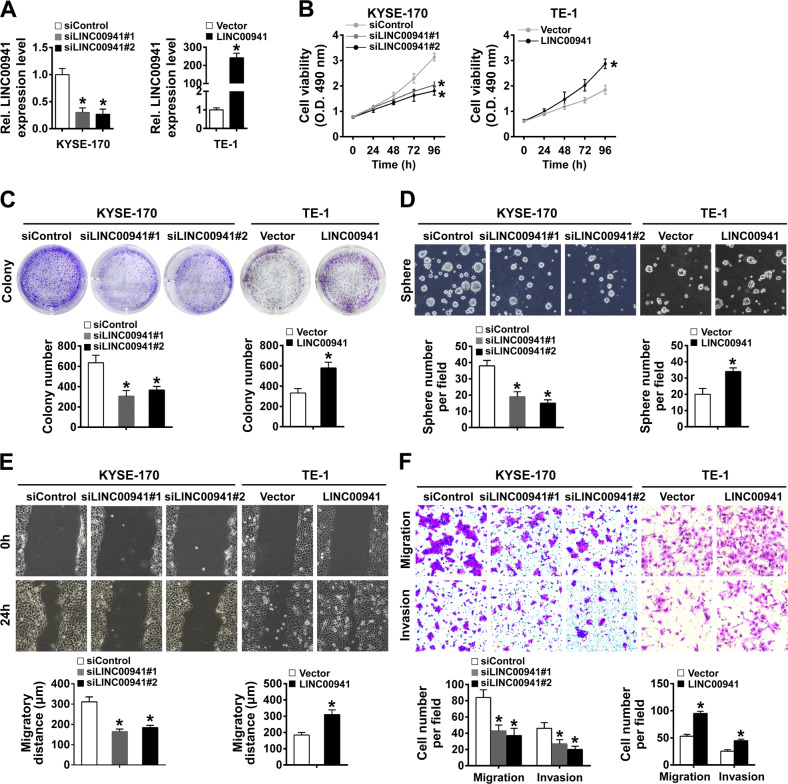
LINC00941 promotes ESCC cells proliferation, stemness, migration, and invasion. **A** LINC00941 expression levels in the indicated cells were measured by RT-qPCR. **B**, **C** The proliferative abilities of the indicated cells were assessed by MTS (**B**) and colony formation assays (**C**). **D** The sphere-formation abilities of the indicated cells were assessed by sphere-formation assay. **E** The wound areas on confluent monolayers of the indicated cells at different time points. **F** The migration and invasion abilities of the indicated cells were assessed by transwell assay. *P < 0.05 compared with control cells. The experiments are representative of three independent experiments with similar results.

To further examine the functional role of LINC00941 in vitro, KYSE-170 cells and TE-1 cells were respectively transfected with two siRNAs targeting LINC00941 or pcDNA3.1-LINC00941 to successfully knockdown or elevate the expression level of LINC00941 (Fig. 2A). As shown in Fig. 2B, C, knockdown of LINC00941 significantly suppressed the proliferation and clone formation rate of KYSE-170 cells detected by MTS and clone formation assays, while overexpression of LINC00941 reached the opposite results in TE-1 cells. Furthermore, the sphere-formation abilities of the siLINC00941 transfected KYSE-170 cells were significantly inhibited, while the LINC00941 overexpressed TE-1 cells were evidently stimulated (Fig. 2D). Moreover, knockdown of LINC00941 significantly suppressed the migration and invasion abilities of KYSE-170 cells detected by wound healing and transwell assays, while overexpression of LINC00941 provided the opposite tendency in TE-1 cells (Fig. 2E, F).

### LINC00941 facilitates tumor growth and metastasis in vivo

**Fig. 3:**
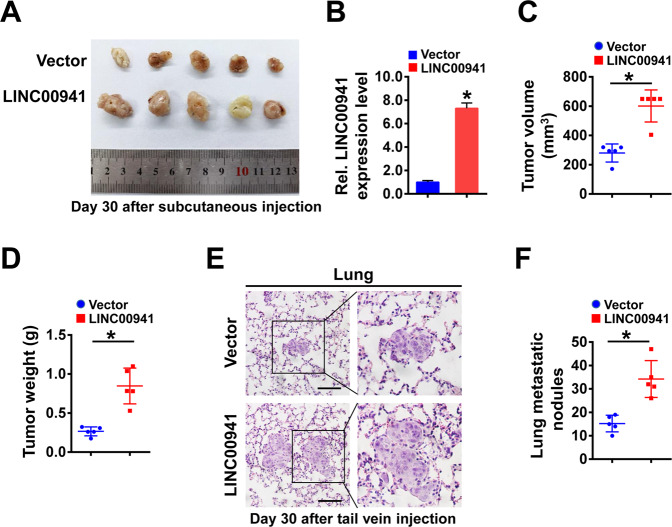
LINC00941 facilitates tumor growth and metastasis in vivo. **A** Image of subcutaneous xenograft tumors following injection of LINC00941-overexpressing TE1 cells (*n* = 5) or control cells (*n* = 5) in BALB/c-nude mice. **B** LINC00941 expression levels in the tumors harvested from mice treated as indicated were measured by RT-qPCR. **C**, **D** The volume (**C**) and weight (**D**) of harvested xenograft tumors were measured. **E** H&E staining in lung sections from BALB/c-nude mice with tail vein injection of LINC00941-overexpressing TE1 cells (*n* = 5) or control cells (*n* = 5). Scale bars, 50 μm. **F** Statistical analysis of the metastatic lung nodules confirmed by H&E staining. *P < 0.05 compared with control mice.

To examine the effect of LINC00941 on tumor growth in vivo, LINC00941-overexpressing TE1 cells or control cells were subcutaneously injected into the flanks of mice. Tumor growth was measured at day 30 after injection. As hypothesized, the tumor volume and tumor weight were significantly increased in mice injected with LINC00941-overexpressing cells than that in control mice (Fig. 3A–D). To evaluate the effect of LINC00941 on tumor metastasis, we intravenously injected LINC00941-overexpressing TE1 cells or control cells into mice via the tail vein. Histological examination by H&E staining identified that the number of lung metastatic nodules formed by LINC00941-overexpressing TE1 cells was evidently increased than that by the control cells (Fig. 3E, F). All of the above data supported that LINC00941 could promote ESCC tumor growth and metastasis.

### Transcription of LINC00941 is regulated by transcription factor SOX2

**Fig. 4:**
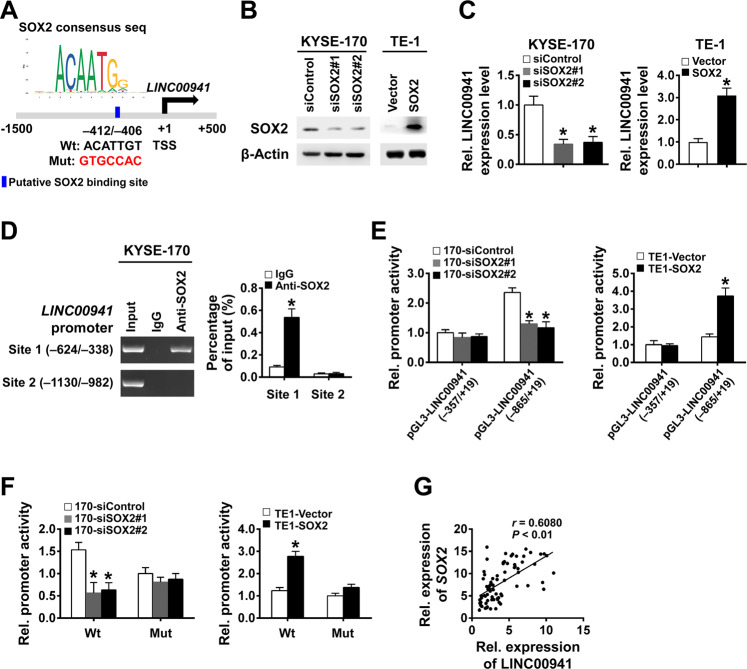
SOX2 transcriptionally activates LINC00941 expression. **A** Schematics show the putative SOX2-binding site on the *LINC00941* promoter region. **B** SOX2 protein levels in the indicated cells were detected by immunoblot. **C** LINC00941 expression levels in the indicated cells were measured by RT-qPCR. **D** The binding of SOX2 on the *LINC00941* promoter containing or lacking putative SOX2-binding element was assessed by ChIP-PCR and ChIP-qPCR assays in KYSE-170 cells transfected with SOX2 expression plasmid using anti-SOX2 antibodies or IgG control. **E** The activity of the *LINC00941* promoter containing or lacking SOX2-binding element was assessed in the indicated cells by a dual-luciferase reporter assay. **F** The activity of wild-type or mutant *LINC00941* promoter was assessed in the indicated cells by a dual-luciferase reporter assay. **G** Pearson correlation analysis was used to analyze the relationship between SOX2 and LINC00941 (*n* = 80). *P < 0.05 compared with control cells. The experiments are representative of three independent experiments with similar results.

Due to the obvious oncogenic effect of LINC00941, we further explored the mechanism affecting LINC00941 transcription and expression. We analyzed the proximal promoter region of *LINC00941* ranging from −1500 to +500 bp relative to transcription start site (TSS) using the online datasets hTFtarget and AnimalTFDB3, and found 80 putative transcription factors possibly bind to the promoter region of *LINC0094*1 (Supplementary Fig. S3A). Among the predicted transcription factors, SOX2 most sparked our interest. Amplification and overexpression of SOX2 represent a hallmark of squamous cancers originating from diverse tissue types and play important roles in stemness maintenance of cancer stem-like cells [15, 16]. Given that LINC00941 promotes stemness of ESCC cells, we then focused on the transcriptionally regulating effect of SOX2 on LINC00941. We identified a potential SOX2-binding element in the *LINC00941* promoter region at −412 ~ −406 bp (Fig. 4A). Two siRNAs targeting SOX2 were then synthesized to knockdown its expression and SOX2 overexpression vector was constructed to successfully elevate its expression (Fig. 4B). Knockdown or overexpression of SOX2 could significantly inhibit or elevate the expression of LINC00941 (Fig. 4C). Further ChIP-PCR and ChIP-qPCR assays in SOX2 overexpressed cells demonstrated the binding effect of SOX2 on the *LINC00941* promoter containing the putative SOX2-binding element (site 1) (Fig. 4D). According to the location of the possible binding site, two fragments containing different promoter regions of *LINC00941* (−357–+19 bp, −865–+19 bp) were cloned into pGL3 basic vector. Significant inhibited luciferase activity of the −865–+19 bp vector in SOX2 knockdown KYSE-170 cells and a promoting effect in SOX2 overexpressed TE-1 cells were then detected by dual-luciferase reporter assay, while no inhibiting or promoting effect was observed in −357–+19 bp vector (Fig. 4E). Further investigation demonstrated no relevant changes on the luciferase activity when the binding site was mutated (Fig. 4F). Moreover, the clinical data confirmed that expression levels of LINC00941 were positively correlated with the expression levels of SOX2 in 80 cases of primary ESCC tumors (Fig. 4G). These results demonstrated that SOX2 positively regulated LINC00941 expression by activating its transcription in ESCC cells.

### LINC00941 directly binds with ILF2 and YBX1

**Fig. 5:**
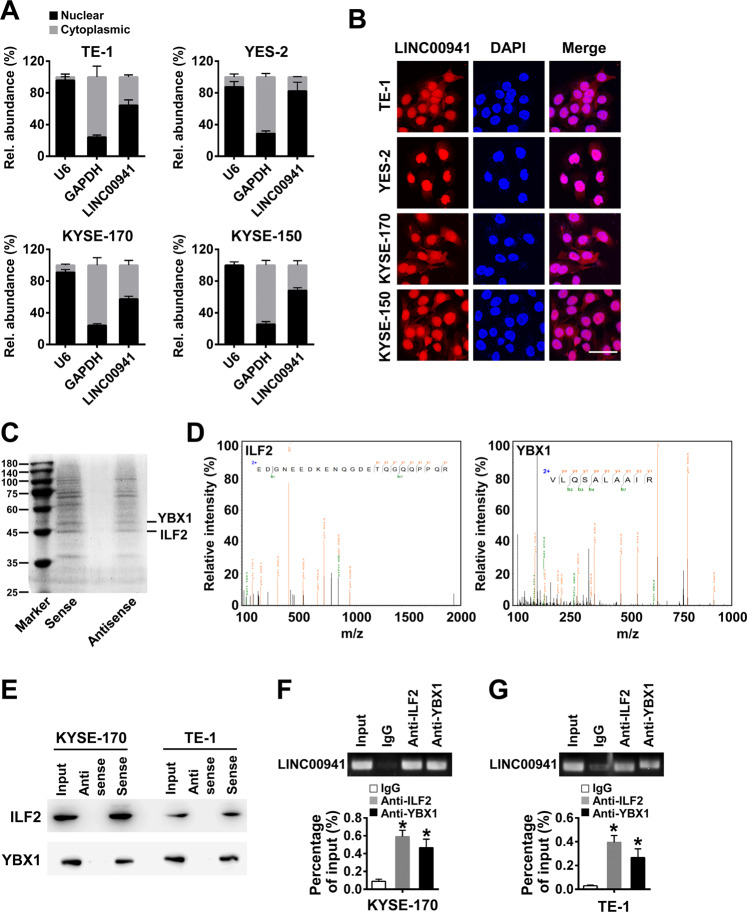
LINC00941 directly interacts ILF2 and YBX1. **A** The subcellular localization of LINC00941 in TE-1, YES-2, KYSE-170, or KYSE-150 cells was measured by qRT-PCR. GAPDH and U6 mRNA were used as controls for cytoplasmic and nuclear transcripts, respectively. **B** The subcellular localization of LINC00941 (red) in TE-1, YES-2, KYSE-170, or KYSE-150 cells was measured by RNA FISH. Nuclei are stained with DAPI. Scale bars, 50 μm. **C** Proteins bound to biotinylated sense or antisense of LINC00941 were separated by SDS-PAGE following RNA pull-down assay and manifested by Coomassie blue staining. **D** Mass spectrometry validated that ILF2 and YBX1 were the binding proteins of LINC00941. **E** The specific interaction of ILF2 or YBX1 with LINC00941 was detected by immunoblot using anti-ILF2 or anti-YBX1 antibodies from RNA pull-down assay in the indicated cells. **F**, **G** Confirmation of the interaction of ILF2 or YBX1 with LINC00941 by RIP assay using anti-ILF2 or anti-YBX1 antibodies in KYSE-170 (**F**) and TE-1 cells (**G**). *P < 0.05 compared with control cells. The experiments are representative of three independent experiments with similar results.

To further uncover the potential mechanisms of LINC00941 in ESCC, we conducted subcellular localization analysis of LINC00941 in ESCC cells and found that LINC00941 was localized both in nucleus and cytoplasm, mainly in nucleus (Fig. 5A). The localization of LINC00941 was further verified by RNA FISH assay (Fig. 5B). RNA pull-down and mass spectrometric analyses were carried out in KYSE170 cells to detect the proteins which LINC00941 could bind with. ILF2 and YBX1 were identified as the bands unique to LINC00941 (Fig. 5C, D). The specific interaction of ILF2 or YBX1 with LINC00941 was further verified by immunoblot and RIP assays in KYSE-170 and TE-1 cells (Fig. 5E–G). ILF2 and YBX1 are master regulator of transcription, RNA splicing and decay through its binding to DNA and RNA. In particular, upregulation of ILF2 and YBX1 has been observed in a growing number of cancers [17–20]. Indeed, we found that the mRNA expression levels of ILF2 and YBX1 were upregulated in esophageal carcinoma tissues compared to normal controls in GEPIA dataset (Supplementary Fig. S4A, B). Although the difference did not reach statistical significance, it represented a strong trend.

### LINC00941 recruits ILF2 and YBX1 onto *SOX2* promoter to activate its expression

**Fig. 6:**
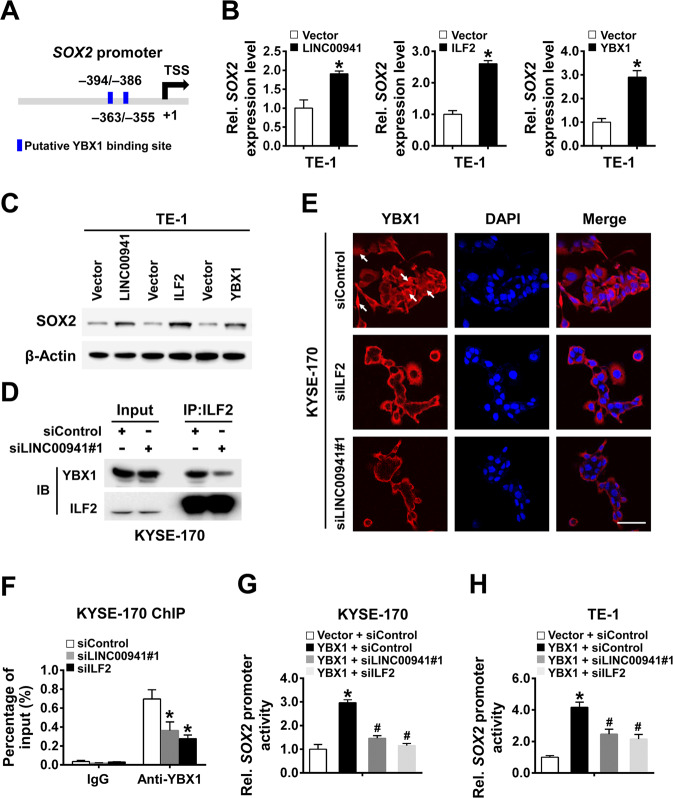
LINC00941 recruits ILF2 and YBX1 onto *SOX2* promoter to activate its expression. **A** Schematics show the putative YBX1-binding site on the *SOX2* promoter region. **B**, **C** The mRNA and protein expression levels of SOX2 were detected by qRT-PCR (**B**) and immunoblot (**C**). **D** The association between ILF2 and YBX1 was detected in the indicated cells by co-immunoprecipitation assay using anti-ILF2 followed by immunoblot with anti-ILF2 or anti-YBX1. **E** The subcellular localization of YBX1 protein was confirmed by immunofluorescence assay in the indicated cells. White arrows indicated representative YBX1 expression in the nucleus. Nuclei are stained with DAPI. Scale bars, 50 μm. **F** The binding of YBX1 on the *SOX2* promoter in the indicated cells was assessed by ChIP-qPCR assay using anti-YBX1 antibodies or IgG control. **G**, **H** The activity of the *SOX2* promoter in the indicated cells was assessed by a dual-luciferase reporter assay. *P < 0.05 compared with the control cells; #*P* < 0.05 compared with YBX1 + siControl. The experiments are representative of three independent experiments with similar results.

Considering the transcriptional regulating function of ILF2 and YBX1 on gene expression, we speculated whether SOX2 was regulated by LINC00941 through ILF2 and YBX1. Indeed, through a sequence search of the *SOX2* promoter, we found potential YBX1-binding elements in the *SOX2* proximal promoter (Fig. 6A). Moreover, overexpression of LINC00941, ILF2, or YBX1 could promote the expression of SOX2 both in mRNA and protein levels (Fig. 6B, C). Because ILF2 is a regulatory cofactor that binds to different gene expression regulation partners at transcriptional level, we proposed that ILF2 may interact with YBX1 to direct SOX2 expression through LINC00941. The binding effect of ILF2 and YBX1 was further verified in KYSE-170 cells by co-immunoprecipitation assay, and this effect could be attenuated by knockdown of LINC00941, further manifesting the indispensable role of LINC00941 in the binding of ILF2 and YBX1 (Fig. 6D). Immunofluorescence assay further demonstrated that the expression of YBX1 protein in the nucleus was weakened by ILF2 or LINC00941 knockdown (Fig. 6E). The binding of YBX1 on the promoter region of *SOX2* was verified by ChIP-qPCR assay, and this binding effect was attenuated by the knockdown of LINC00941 or ILF2 (Fig. 6F). Moreover, significant elevated luciferase activity of the *SOX2* promoter was detected in YBX1 overexpressed KYSE-170 and TE1 cells, and the effect was eliminated accompanied by knockdown of LINC00941 or ILF2 (Fig. 6G, H), suggesting the important and indispensable role of LINC00941/YBX1/ILF2 complex in regulating the transcription of *SOX2*.

### LINC00941 functions as a scaffold for ILF2 and YBX1 to promote mRNA stability of SOX2

**Fig. 7:**
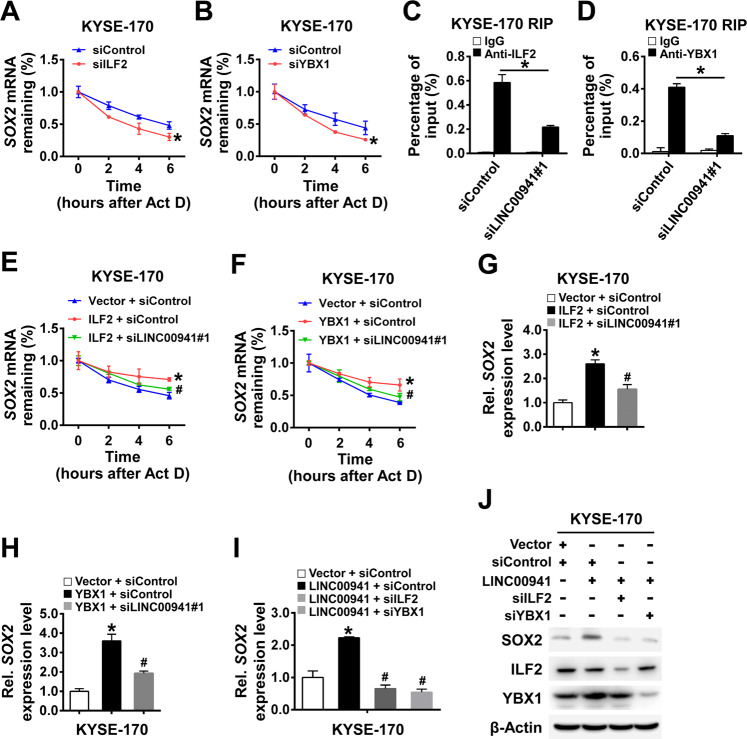
LINC00941 functions as a scaffold for ILF2 and YBX1 to promote mRNA stability of SOX2. **A**, **B** The stability of *SOX2* mRNA was detected by qRT-PCR at the different actinomycin D (5 µg/mL) treatment times in the indicated cells. **C**, **D** The interaction of ILF2 or YBX1 with *SOX2* mRNA was detected by RIP assay using anti-ILF2 or anti-YBX1 antibodies in the indicated cells. **E**, **F** The stability of *SOX2* mRNA was detected by qRT-PCR in the indicated cells treated with actinomycin D (5 µg/mL). **G**–**I** The expression levels of *SOX2* in the indicated cells were detected by qRT-PCR. **J** The protein levels of SOX2, ILF2, and YBX1 were detected by immunoblot. *P < 0.05 compared with the control cells; #, *P* < 0.05 compared with ILF2 + siControl (**E**, **G**), YBX1 + siControl (**F**, **H**), LINC00941 + siControl (**I**) group, respectively. The experiments are representative of three independent experiments with similar results.

ILF2 and YBX1 drive resistance to DNA-damaging agents by binding mRNA to influence the splicing and stabilization of mRNA [17]. ILF2 also facilitates nuclear mRNA export and inhibits hMTR4-mediated exosomal degradation to promote stabilization and expression of SOX2, NANOG, and SALL4 [18]. Therefore, we then tested the influence of LINC00941 binding with ILF2 and YBX1 on mRNA stability of *SOX2*. Knockdown of ILF2 or YBX1 shortened the half-life of *SOX2* mRNA in actinomycin D treated cells, suggesting the role of ILF2 and YBX1 in maintaining stability of *SOX2* mRNA (Fig. 7A, B). Importantly, ILF2 and YBX1 were detected to be abundantly enriched on the mRNA of *SOX2* by RIP assay, but this phenomenon was eliminated in the absence of LINC00941, suggesting the indispensable role of LINC00941 (Fig. 7C, D). As expected, overexpression of ILF2 or YBX1 significantly prolonged the half-life of *SOX2* mRNA; however, the effect was markedly reversed in the absence of LINC00941 (Fig. 7E, F). Likewise, knockdown of LINC00941 effectively reduced the ILF2- or YBX1-mediated increase of *SOX2* mRNA (Fig. 7G, H). These results suggested that LINC00941 may increase mRNA stability of *SOX2* through interacting with ILF2 and YBX1.

Furthermore, the enhanced mRNA and protein expression level of SOX2 caused by overexpression of LINC00941 was substantially blocked by knockdown of ILF2 or YBX1 (Fig. 7I, J). Taken together, our results suggested that LINC00941 could upregulate SOX2 through interacting with ILF2 and YBX1 at the transcriptional and post-transcriptional levels.

### SOX2 mediates the LINC00941-regulated promotion of ESCC progression

**Fig. 8:**
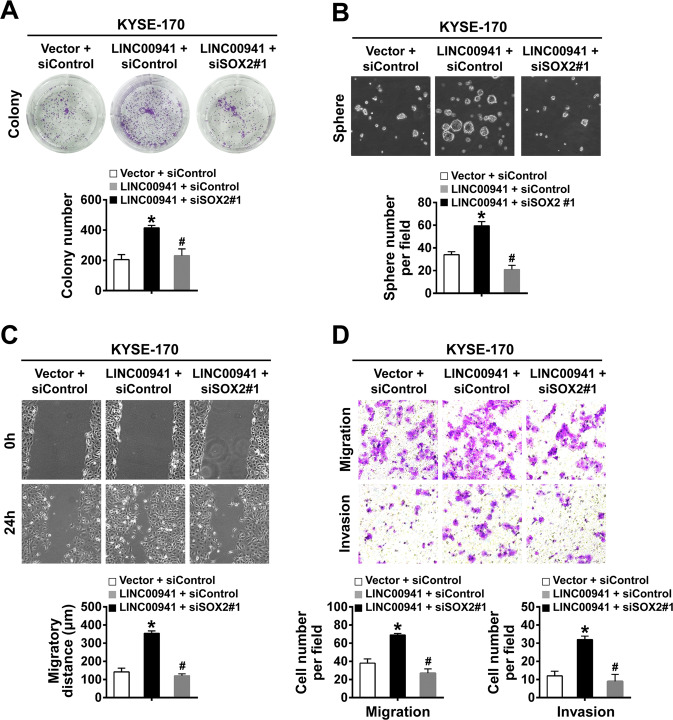
SOX2 mediates the LINC00941-regulated promotion of ESCC progression. **A** The proliferative abilities of the indicated cells were assessed by colony formation assay. **B** The sphere-formation abilities of the indicated cells were assessed by sphere-formation assay. **C** The wound areas on confluent monolayers of the indicated cells at different time points. **D** The migration and invasion abilities of the indicated cells were assessed by transwell assay. *P < 0.05 compared with the control cells; #*P* < 0.05 compared with LINC00941 + siControl group. The experiments are representative of three independent experiments with similar results.

We further performed cell-functional assays to uncover whether SOX2 mediated the LINC00941-regulated promotion of ESCC progression. As shown in Fig. 8A, the enhanced colony formation rate of KYSE-170 cells caused by overexpression of LINC00941 was blocked by knockdown of SOX2. Furthermore, the elevated sphere-formation abilities of KYSE-170 cells caused by LINC00941 overexpression were violently weakened by SOX2 knockdown (Fig. 8B). Knockdown of SOX2 also reversed the enhanced migration and invasion ability of KYSE-170 cells caused by overexpression of LINC00941 (Fig. 8C, D). These results suggested that SOX2 mediated LINC00941-regulated promotion of ESCC progression.

## Discussion

LINC00941 is a novel lncRNA that has been found to exhibit protumorigenic and prometastatic behaviors during tumorigenesis. Although LINC00941 was recently reported to regulate ESCC progression via functioning as a competing endogenous RNA (ceRNA) for miR-877-3p to modulate PMEPA1 expression [12], the functional significance of LINC00941 in ESCC is far from clear. In the present study, we observed that LINC00941 was highly expressed in ESCC and transactivated by SOX2. Furthermore, LINC00941 could bind RNA/DNA-binding proteins ILF2 and YBX1, and potentiate SOX2 expression both at the transcriptional and post-transcriptional levels. Our data revealed that LINC00941 coordinated with SOX2 to form a positive feed-forward loop to drive tumorigenesis of ESCC, thus providing a rationale to target LINC00941 as a novel therapeutic strategy.

Upregulation of LINC00941 has been detected in several types of cancers. Overexpression of LINC00941 promoted the progression of gastric cancer (GC) via regulating cancer-related biological processes [21, 22]. The expression of LINC00941 was elevated in lung adenocarcinoma (LUAD) and associated with phosphorylation of the PI3K-AKT signaling pathway [23]. The overexpression of LINC00941 in ESCC tissues and cell lines was detected in the present study and Zhang’s study [12], together with the promoting effect on ESCC cells proliferation, migration, and invasion, suggesting the oncogenic role of LINC00941 in ESCC progression. However, due to the diversity of the functional mechanisms of lncRNA, the specific mechanisms of LINC00941 in ESCC remains to be further investigated.

By searching the online datasets, we noticed the possible binding site of SOX2 in the promoter region of LINC00941. SOX2 is a member of the SRY-related HMG-box (SOX) family of transcription factors involved in the regulation of embryonic development and in the determination of cell fate. Based on the following consideration, we selected SOX2 for our further study. Firstly, among the predicted transcription factors, SOX2 is an amplified lineage-survival oncogene in ESCC [24]. Secondly, SOX2 is a core regulator that determines chromatin accessibility, epigenetic modifications, and gene expression patterns in ESCC cells [25]. Our most recent studies also validate that SOX2 is significantly upregulated in ESCC, and SOX2 transcriptionally regulate the expression of NRSN2-AS1 and KTN1-AS1, which promote ESCC cells proliferation, migration, invasion, and epithelial-mesenchymal transition (EMT) [26, 27]. Finally, we detected the evident promoting role of LINC00941 in sphere formation abilities of ESCC cells and this effect prompt us to explore the regulatory mechanism between SOX2 and LINC00941. In the present study, our findings suggested that transcription of LINC00941 was activated by SOX2 to promote the progression and stemness maintenance of ESCC cells. Of note, the conserved DNA-binding motif “ACAATGG” of SOX2 is not specific. The core sequence of SOX2, SOX4, SOX8, SOX9, SOX13, and SOX15 binding motif predicted by JASPAR (http://jaspar.genereg.net/) show the largest degree of similarity. Therefore, we speculated that SOX4, SOX8, SOX9, SOX13, and SOX15 may also transcriptionally bind LINC00941. This inference should be validated by further experiments.

LncRNA exerts its roles dependent on its subcellular localization. Zhang’s study reported the role of LINC00941 as ceRNA in cytoplasm, which it acted as a ceRNA for miR-877-3p to modulate the expression of PMEPA1 to further regulate the EMT process in ESCC cells [12]. The ceRNA role of LINC00941 was also reported in pancreatic cancer [28] and hepatocellular carcinoma [29]. We detected the localization of LINC00941 both in nucleus and cytoplasm of ESCC cells, so we considered that LINC00941 could exert its roles both in nucleus and cytoplasm in ESCC. We revealed that LINC00941 could bind with ILF2 and YBX1. ILF2, also known as nuclear factor 45 (NF45), is encoded by a gene located on human chromosome 1q21.3. ILF2 was first identified to be associated with ILF3/NF90 in the nucleus to regulate IL-2 gene transcription at the antigen receptor response element (ARRE)/nuclear factor of activated T-cells (NFAT) DNA target sequence [30]. Further investigations confirmed that ILF2/ILF3 complex could affect the redistribution of nuclear mRNA to the cytoplasm and mRNA stabilization, repair DNA breaks by nonhomologous end joining, and negatively regulate the microRNA processing pathway. Overexpression of ILF2 was frequently observed in non-small cell lung cancer, glioma, childhood endodermal sinus tumors, ESCC, and hepatocellular carcinoma [31–35]. Furthermore, ILF2 was recently reported to interact with YBX1, a broad RNA binding protein, to modulate DNA damage-induced splicing regulation [17]. YBX1 functions as both a DNA and RNA binding protein and its aberrant expression is associated with cancer proliferation and invasion in numerous tumor tissues [19]. Some lncRNAs, such as LINC02042 [20], HOXC-AS3 [36], and LINC00312 [37] have been identified to interact with YBX1 to enhance mRNA stability. In the present study, LINC00941 could not affect the expression level of ILF2 and YBX1, but rather recruited ILF2 and YBX1 to form LINC00941/ILF2/YBX1 complex to influence SOX2 expression both in transcriptional and post-transcriptional levels. One side, LINC00941 recruited ILF2 and YBX1 to the mRNA of SOX2 to enhance its stabilization, the other side, LINC00941 recruited ILF2 and YBX1 to the promoter region of SOX2 to promote its transcription. Besides, silencing of SOX2 blocked the enhanced cell phenotype caused by LINC00941 overexpression, indicating that SOX2 mediated LINC00941-regulated promotion of ESCC progression.

Taken together, our study reveals the upregulation and oncogenic role of LINC00941 in ESCC. The transcription of LINC00941 is activated by SOX2, meanwhile, LINC00941 binds with ILF2 and YBX1 to increase the mRNA stabilization and promote transcription of SOX2. LINC00941 may be act as a promising novel biomarker and therapeutic target for patients with ESCC.

## Supplementary information

Figure S1

Figure S2

Figure S3

Figure S4

Supplementary Figure Legends

Supplementary Tables

Original western blots

Reporting Checklist

## Data Availability

The data supporting this study are available from the corresponding author upon request.
