# Supplementary figures and images for: Reciprocal regulation of LINC00941 and SOX2 promotes progression of esophageal squamous cell carcinoma
Source: Cell Death Dis. 2023 Jan 30;14(1):72. doi: 10.1038/s41419-023-05605-6 (PMC9886991; doi:10.1038/s41419-023-05605-6)

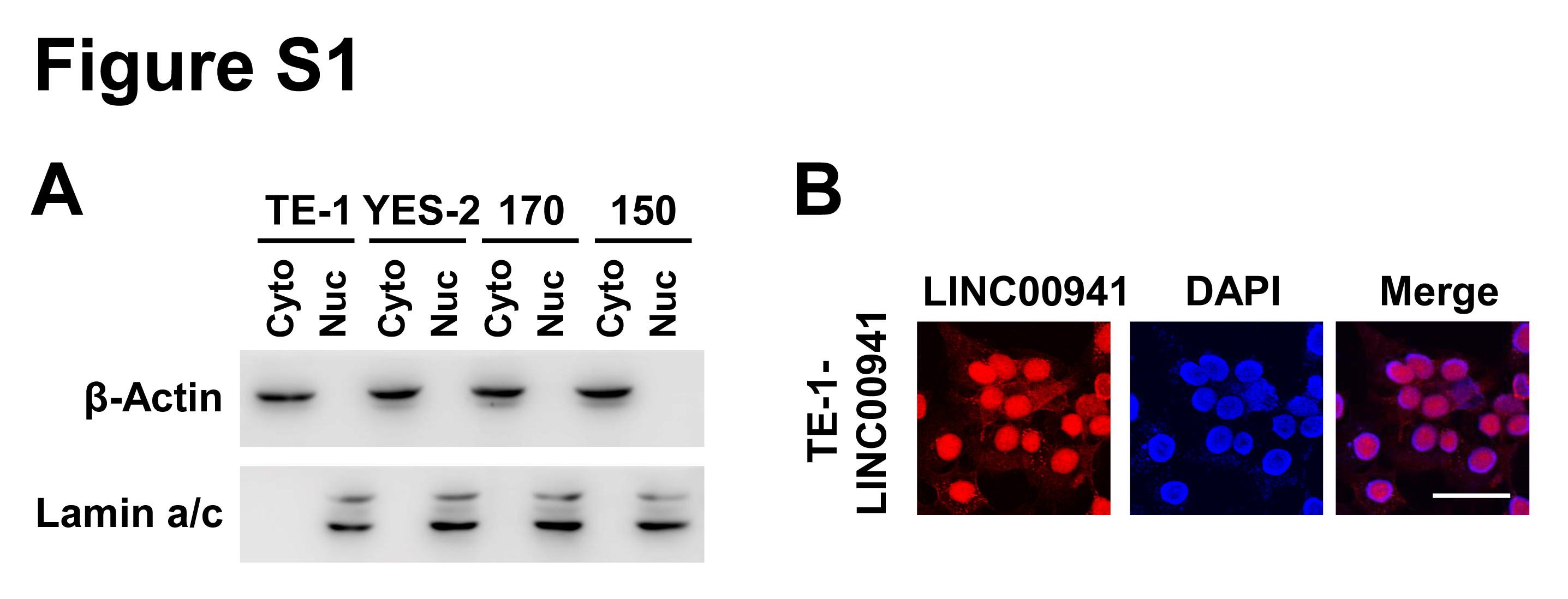

Supplement: Supplementary file 1 — Figure S1 [file 41419_2023_5605_MOESM1_ESM.tif]

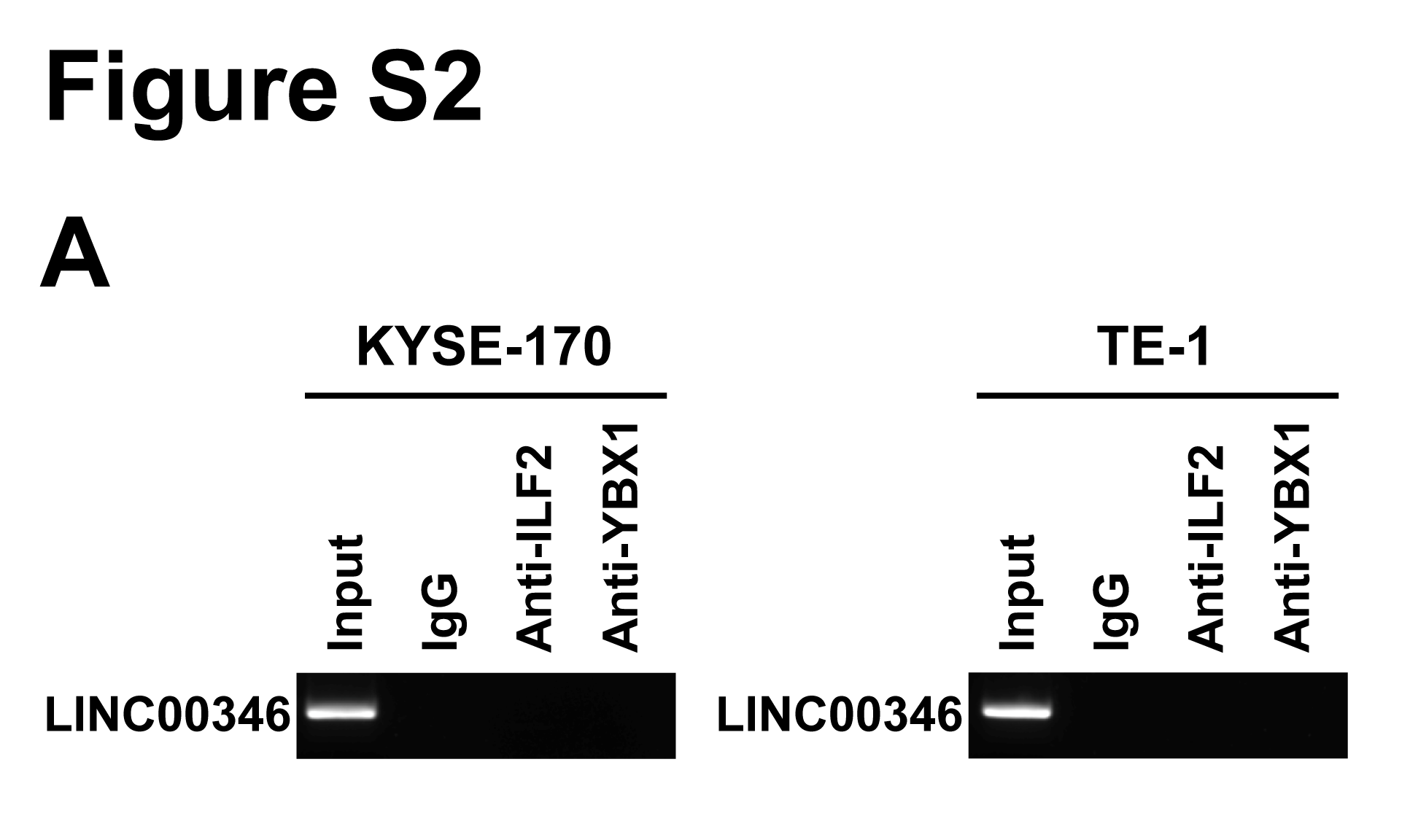

Supplement: Supplementary file 2 — Figure S2 [file 41419_2023_5605_MOESM2_ESM.tif]

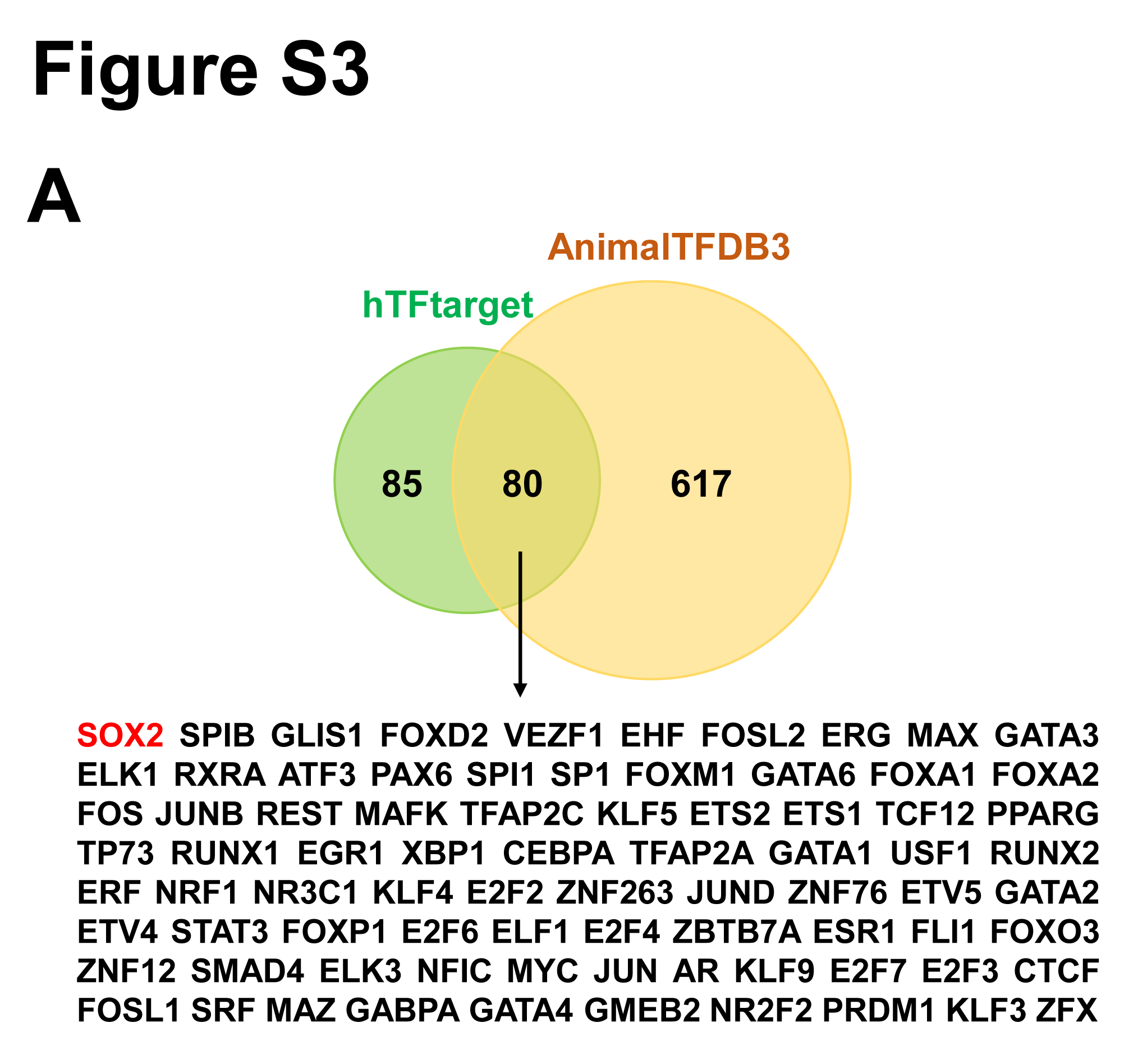

Supplement: Supplementary file 3 — Figure S3 [file 41419_2023_5605_MOESM3_ESM.tif]

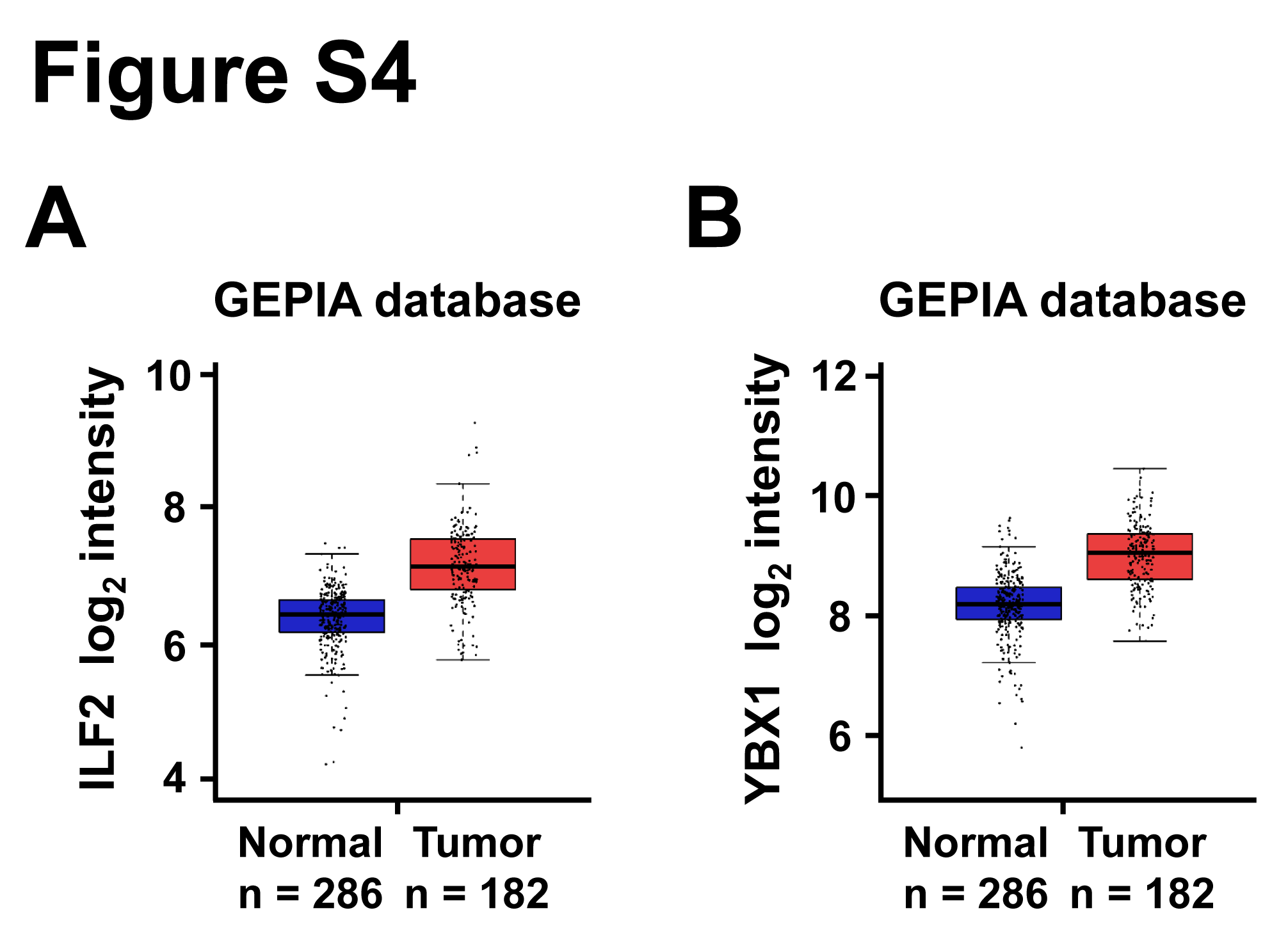

Supplement: Supplementary file 4 — Figure S4 [file 41419_2023_5605_MOESM4_ESM.tif]
