# Supplementary material for: Reciprocal regulation of LINC00941 and SOX2 promotes progression of esophageal squamous cell carcinoma
Source: Cell Death Dis. 2023 Jan 30;14(1):72. doi: 10.1038/s41419-023-05605-6 (PMC9886991; doi:10.1038/s41419-023-05605-6)
Supplement: Supplementary file 5 — Supplementary Figure Legends [file 41419_2023_5605_MOESM5_ESM.docx]

**Supplementary Figure Legends**

**Figure S1. LINC00941 was localized both in nucleus and cytoplasm, mainly in nucleus. A** The separation of nucleus and cytoplasm was estimated by western blot. Lamin A/C was used as the nucleus marker and β-Actin as the cytoplasm marker. **B** The subcellular localization of LINC00941 (red) in LINC00941 overexpressed TE-1 cells was measured by RNA FISH. Nuclei are stained with DAPI. Scale bars, 50 μm.

**Figure S2.** The interaction of ILF2 or YBX1 with LINC00346 (an unrelated lncRNA) was detected by RIP assay using anti-ILF2 or anti-YBX1 antibodies in KYSE-170 and TE-1 cells.

**Figure S3.** Venn diagram of putative transcription factors which might bind to the *LINC00941* promoter from two different online datasets.

**Figure S4.** Expression levels of ILF2 and YBX1 in 182 esophageal carcinoma (ESCA) samples and 286 normal controls from GEPIA cohort.
