# Supplementary material for: Reciprocal regulation of LINC00941 and SOX2 promotes progression of esophageal squamous cell carcinoma
Source: Cell Death Dis. 2023 Jan 30;14(1):72. doi: 10.1038/s41419-023-05605-6 (PMC9886991; doi:10.1038/s41419-023-05605-6)
Supplement: Supplementary file 6 — Supplementary Tables [file 41419_2023_5605_MOESM6_ESM.docx]

**Supplementary Tables**

**Table 1. Clinicopathological features and clinical data of 80 ESCC patients.**

| Clinicopathological features |  | Number (80) |
| --- | --- | --- |
| Age  ≤60  >60  Gender  Male  Female  Depth of tumor invasion  T1+T2  T3+T4  Lymph node metastasis  Negative  Positive  TNM stage  I+II  III+IV  Differentiation grade  Well/Moderate  Poor | |  |
|  |  | 33 (41.25%) |
|  |  | 47 (58.75%) |
|  |  |  |
|  |  | 45 (56.25%) |
|  |  | 35 (43.75%) |
|  |  |  |
|  |  | 30 (37.5%) |
|  |  | 50 (62.5%) |
|  |  |  |
|  |  | 29 (36.25%) |
|  |  | 51 (63.75%) |
|  |  |  |
|  |  | 36 (45%) |
|  |  | 44 (55%) |
|  |  |  |
|  |  | 53 (66.25%) |
|  |  | 27 (33.75%) |

**Table 2. The siRNAs targeting LINC00941, SOX2, ILF2, and YBX1**

| **Target gene** | **siRNA sequence or**  **commercial reference number** |
| --- | --- |
| siLINC00941#1 | [siG161205034859-1-5](https://www.ribobio.com/view_product.php?sku=siG161205034859-1-5) (RiboBio Co. Ltd) |
| siLINC00941#2 | [siG161205034908-1-5](https://www.ribobio.com/view_product.php?sku=siG161205034908-1-5) (RiboBio Co. Ltd) |
| siSOX2#1 | CTGCAGTACAACTCCATGA |
| siSOX2#2 | CTGCCGAGAATCCATGTATAT |
| siILF2 | GCTATCTTGCTTCTGAAATAT |
| siYBX1 | GGACGGCAATGAAGAAGAT |

**Table 3. Primer sequences used for RT-qPCR assays**

| **Gene** | **Sequence (5'-3')** | | **Product size (bp)** |
| --- | --- | --- | --- |
| *LINC00941* | Forward | GCATACTGACAATACAAACCGAA | 142 |
|  | Reverse | ATCAATTCAAATCAAGAGCCCAA |  |
| *SOX2* | Forward | TCCCATCCACACTCACGCAAA | 207 |
|  | Reverse | TGCAAAGCTCCTACCGTACCAC |  |
| *GAPDH* | Forward | AGGTGAAGGTCGGAGTCAACG | 102 |
|  | Reverse | AGGGGTCATTGATGGCAACA |  |
| *U6* | Forward | CTCGCTTCGGCAGCACA | 94 |
|  | Reverse | AACGCTTCACGAATTTGCGT |  |

**Table 4. Primers for ChIP-qPCR and dual-luciferase reporter assays**

| **Gene** | **Region on promoter** | **Forward primer** | | **Reverse primer** |
| --- | --- | --- | --- | --- |
| **Primers for ChIP-qPCR** | | |  | |
| *LINC00941* | –624/–338 | TCCTGCTAAATAGACACACTCTC | | TTGCTGTGAGCCAGGACCAT |
| *LINC00941* | –1130/–982 | CAGAGACAGGGTTTCATCGT | | ACTTCATAGAAAAGGCATCTGGG |
| *SOX2* | –485/–238 | ATGAGCGGGAGAACAATGACACA | | CAGGTCACACCACACGCCTT |
| **Primers for the construction of luciferase reporters** | | | | |
| *LINC00941* | –865/+19 | GAAGCTAGCCCTCCGAAATGAGTTAGGTCCC | | GACAAGCTTTGACTTCAGCCACGTCCCC |
| *LINC00941* | –357/+19 | GAAAGATCTATGGTCCTGGCTCACAGCAA | | GACAAGCTTTGACTTCAGCCACGTCCCC |
| *SOX2* | –480/+224 | GAAGCTAGCCGGGAGAACAATGACACACCAAC | | GACAAGCTTTCCTCCTCTGGCCGATCCTG |

**Table 5. Antibodies used for immunoblot and immunofluorescence**

| **Antibody** | **Source** | **Company** | **Catalogue number** | **Dilution** |
| --- | --- | --- | --- | --- |
| β-Actin | Rabbit | Zenbio | 380624 | IB 1:5000 |
| Lamin A/C | Mouse | Zenbio | 201015-3D6 | IB 1:1000 |
| SOX2 | Rabbit | Cell signaling technology | D9B8N | IB 1:1000  ChIP 1:50 |
| ILF2 | Rabbit | Zenbio | 382994 | IB 1:1000  IP 1:50 |
| YBX1 | Mouse | Abcam | Ab76149 | IB 1:1000  IF 1:100  IP 1:50  ChIP 1:50 |

IB: Immunoblot; IF: Immunofluorescence; ChIP: Chromatin immunoprecipitation

**Table 6. LINC00941-bound proteins with top 50 matching scores**

**identified by mass spectrometry**

| **Name** | **Accession** | **Protein name** |
| --- | --- | --- |
| 1  2  3  4  5  6  7  8  9  10  11  12  13  14  15  16  17  18  19  20  21  22  23  24  25  26  27  28  29  30  31  32  33  34  35  36  37  38  39  40  41  42  43  44  45  46  47  48  49  50 | P62318  A6NLN1  A0A6I8PL42  Q7Z2V5  P46783  H0Y8X0  P11908  Q15393  P52565  B5BU25  O43684  P51116  P51991  P50995  P59827  Q16629  A0A0B4J259  A0A0J9YYL3  F6RFD5  P00441  Q6P1N4  O43390  A0A024RA87  H3BND3  P0DN76  H3BPK7  K7EJV9  Q5JX56  F8WE72  P62266  P20290  P28074  P13798  P35998  H0YF90  J3KQN4  Q3B7A7  Q32Q75  A0A6Q8PFC1  P13667  A0A3B3ITK7  P67809  B4DY09  Q08211  B2R959  Q15046  P05198  Q12906  B4DUQ1  B2R8Z8 | SNRPD3  PTBP1  LARS1  DKFZp686J01190  RPS10  DEK  PRPS2  SF3B3  ARHGDIA  U2AF2  BUB3  FXR2  HNRNPA3  ANXA11  BPIFB4  SRSF7  LYZ  PUF60  DSTN  SOD1  IQGAP1  HNRNPR  SEPT7  NUDT21  U2AF1L5  AARS1  RPL23A  CPNE1  SBDS  RPS23  BTF3  PSMB5  APEH  PSMC2  M6PR  RPL36A  GART  EIF4E  YARS1  PDIA4  PGM1  YBX1  ILF2  DHX9  HNRPL  KARS1  EIF2S1  ILF3  HNRNPU  SYNCRIP |
