# Supplementary material for: Reciprocal regulation of LINC00941 and SOX2 promotes progression of esophageal squamous cell carcinoma
Source: Cell Death Dis. 2023 Jan 30;14(1):72. doi: 10.1038/s41419-023-05605-6 (PMC9886991; doi:10.1038/s41419-023-05605-6)
Supplement: Supplementary file 7 — Original western blots [file 41419_2023_5605_MOESM7_ESM.docx]

Figure 4B

**β-Actin**


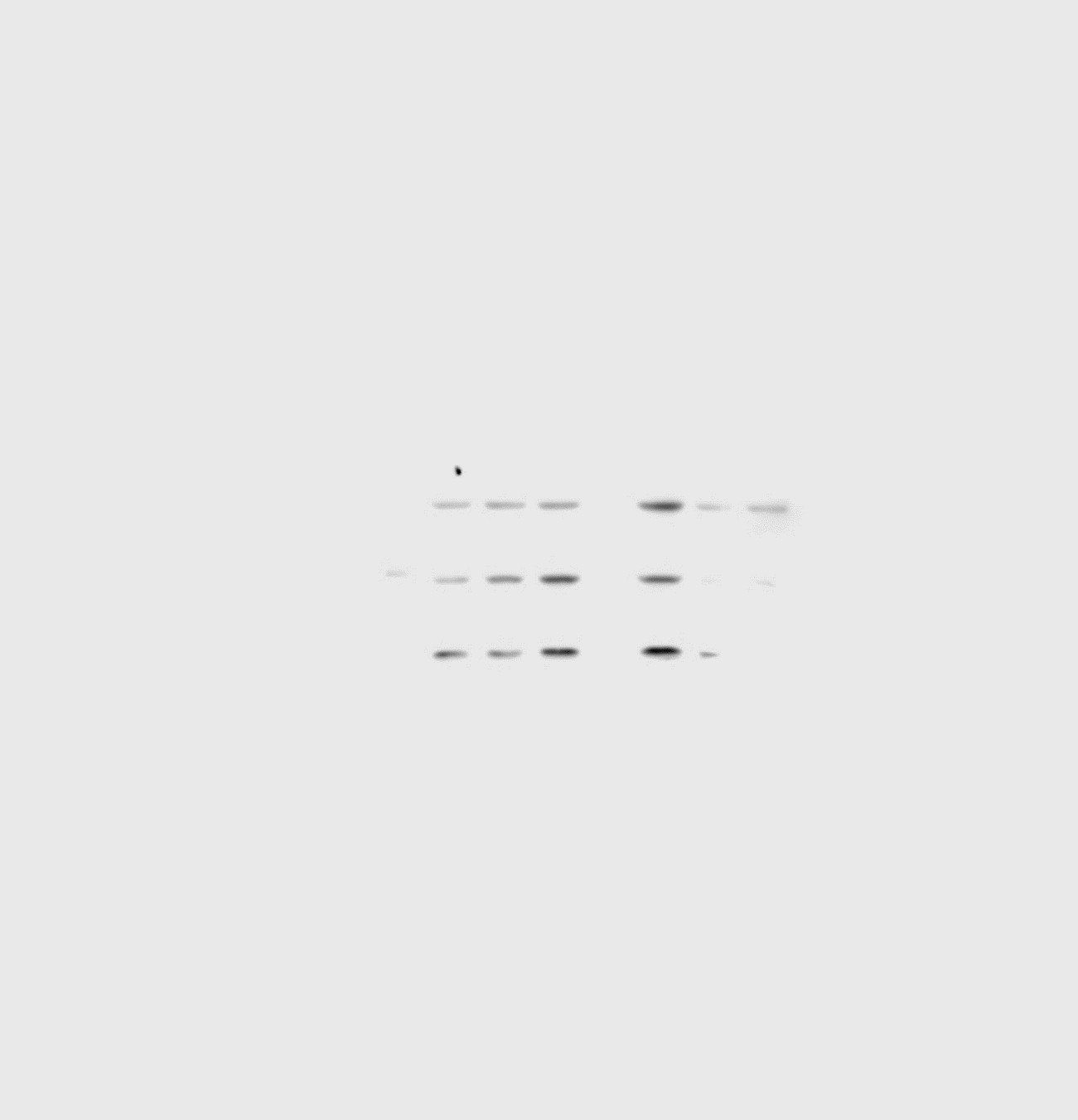


**SOX2**


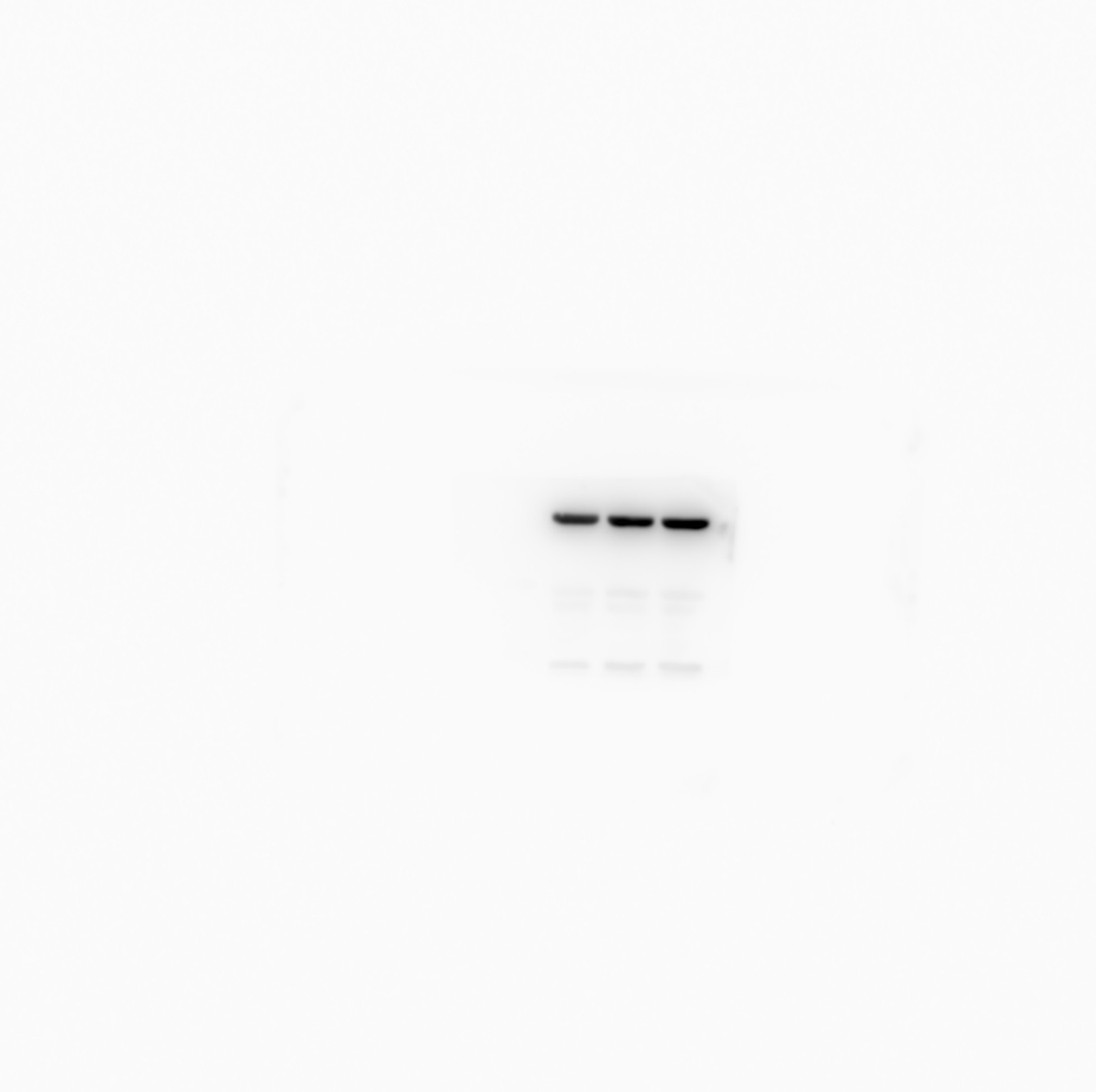


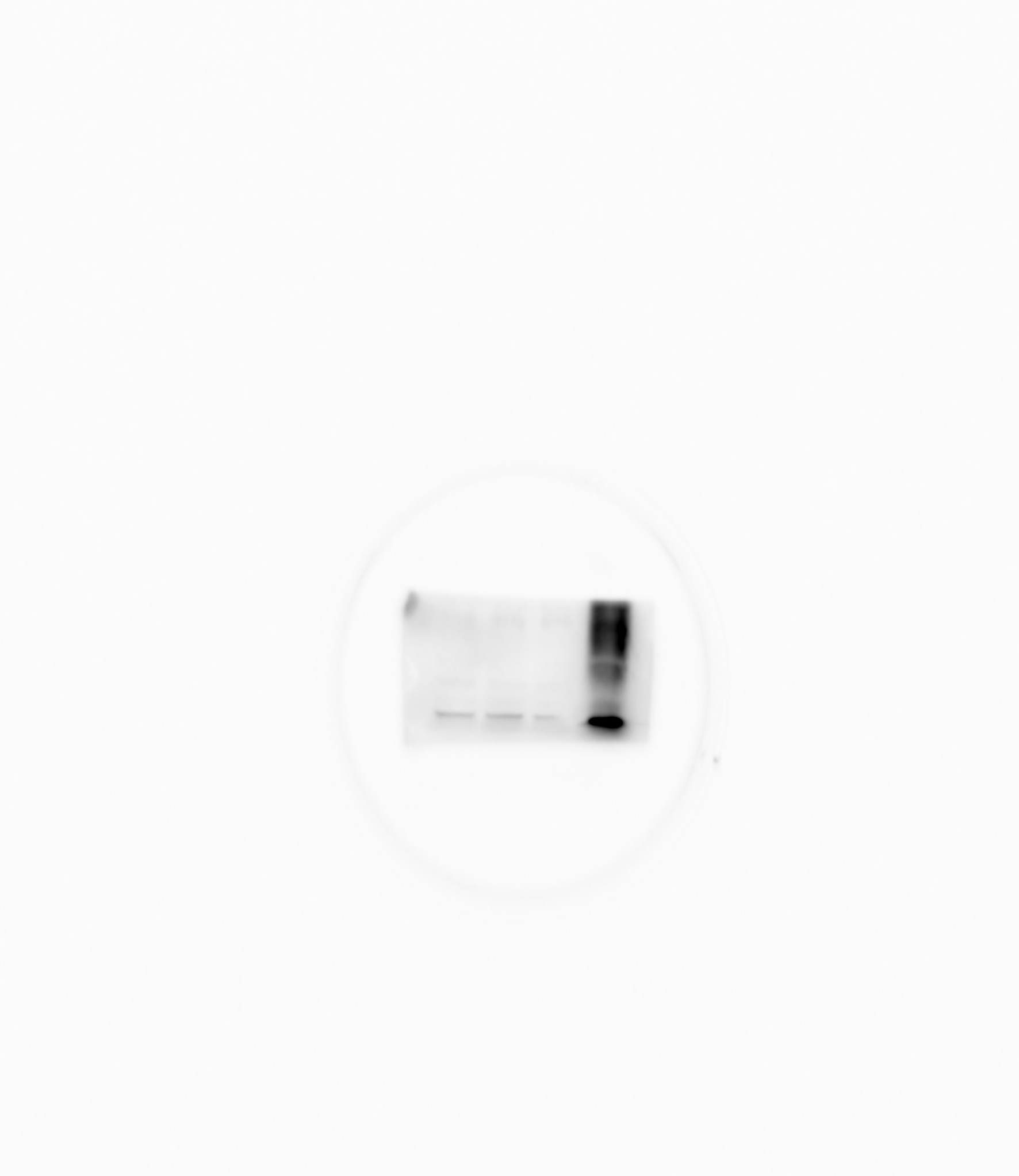


**SOX2**


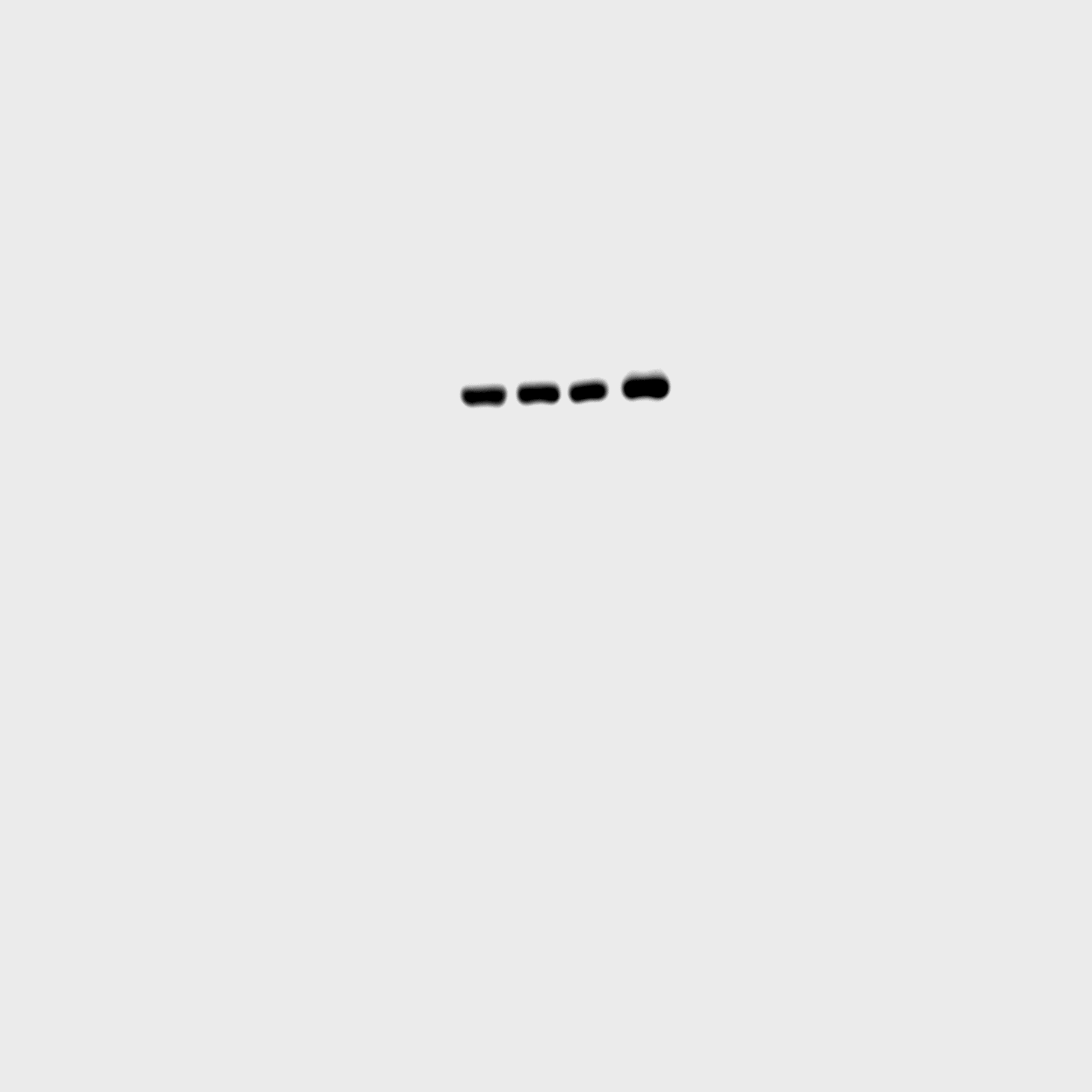


**β-Actin**


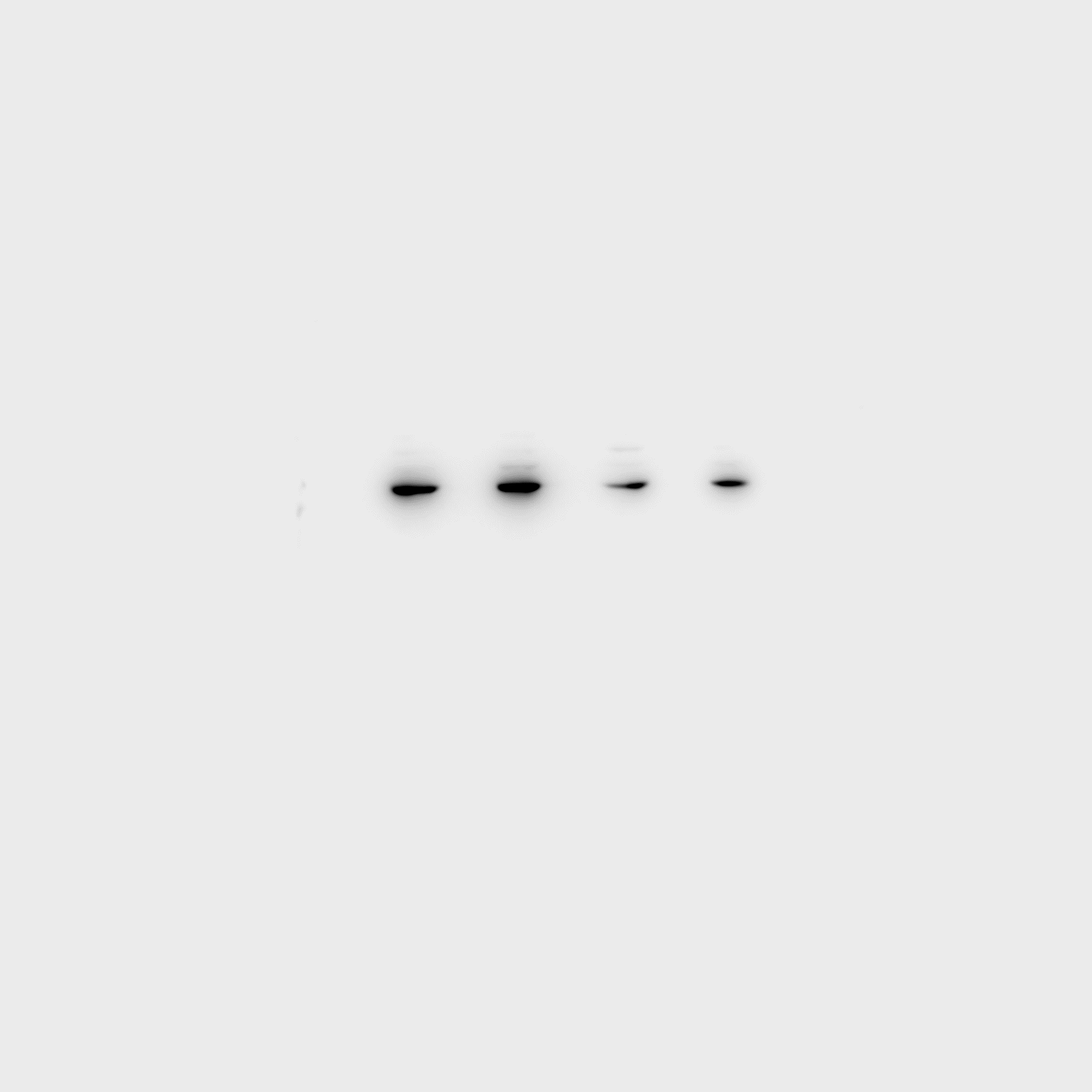
Figure 5E

**YBX1**


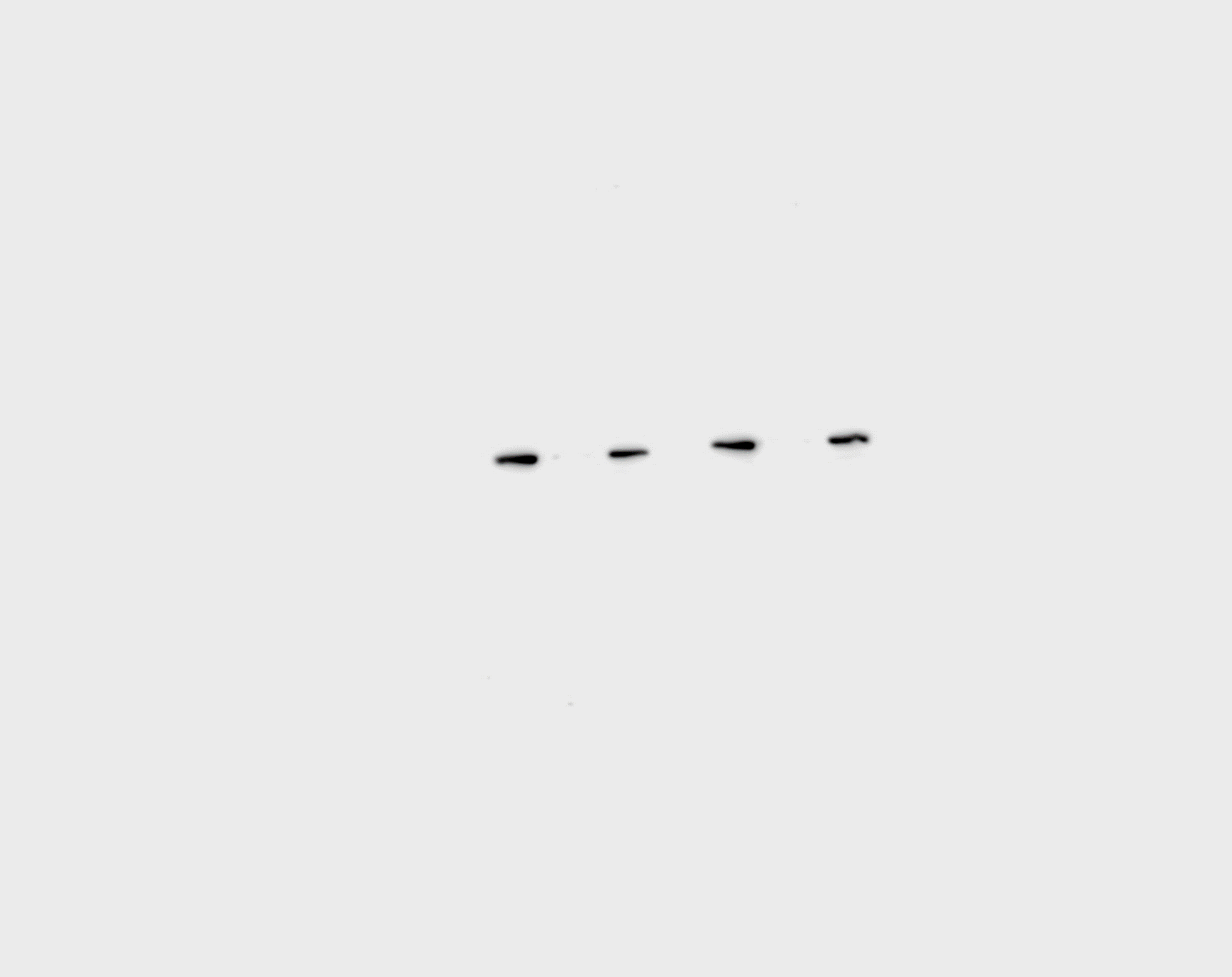


**ILF2**

Figure 6C


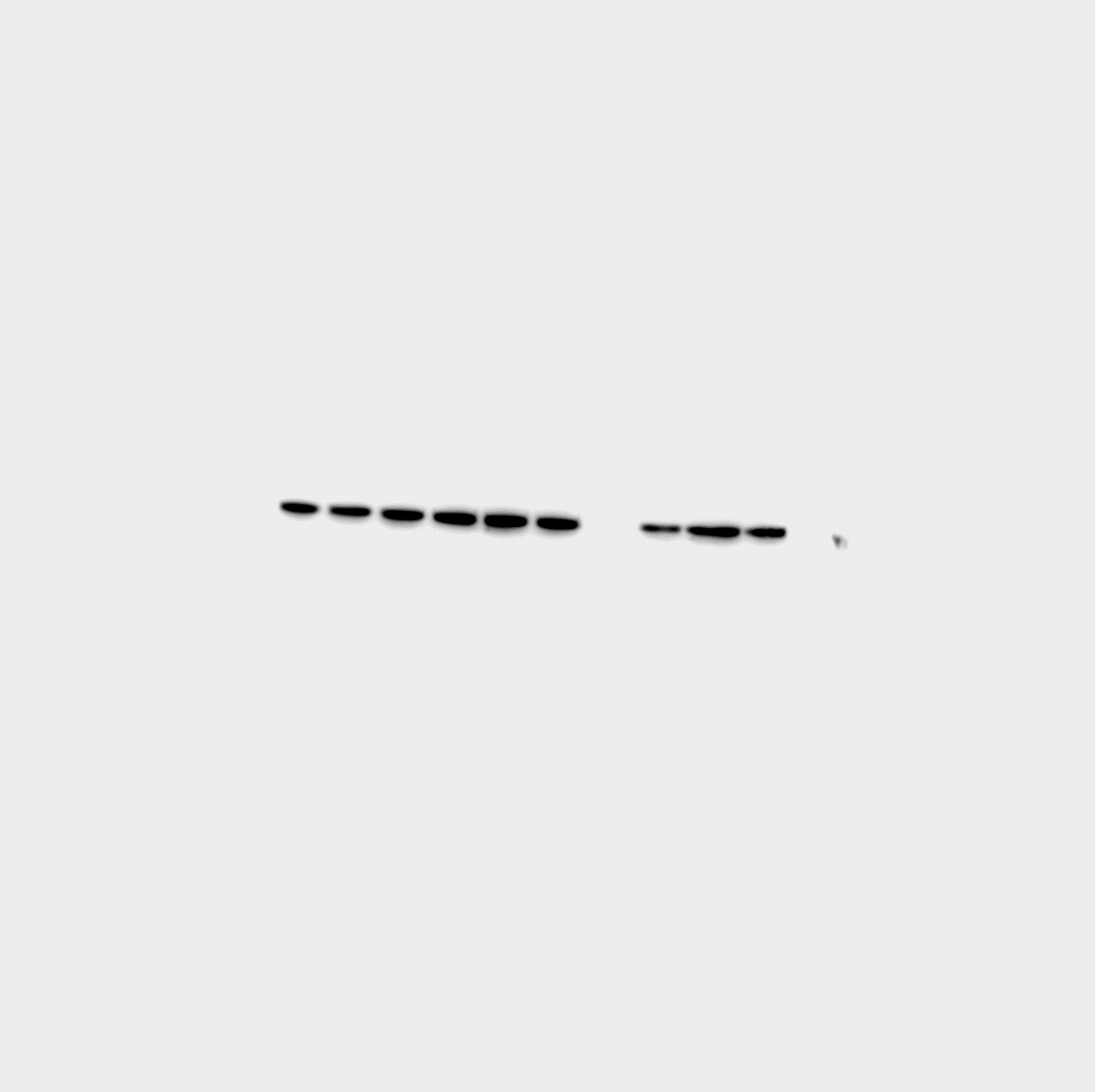


**β-Actin**


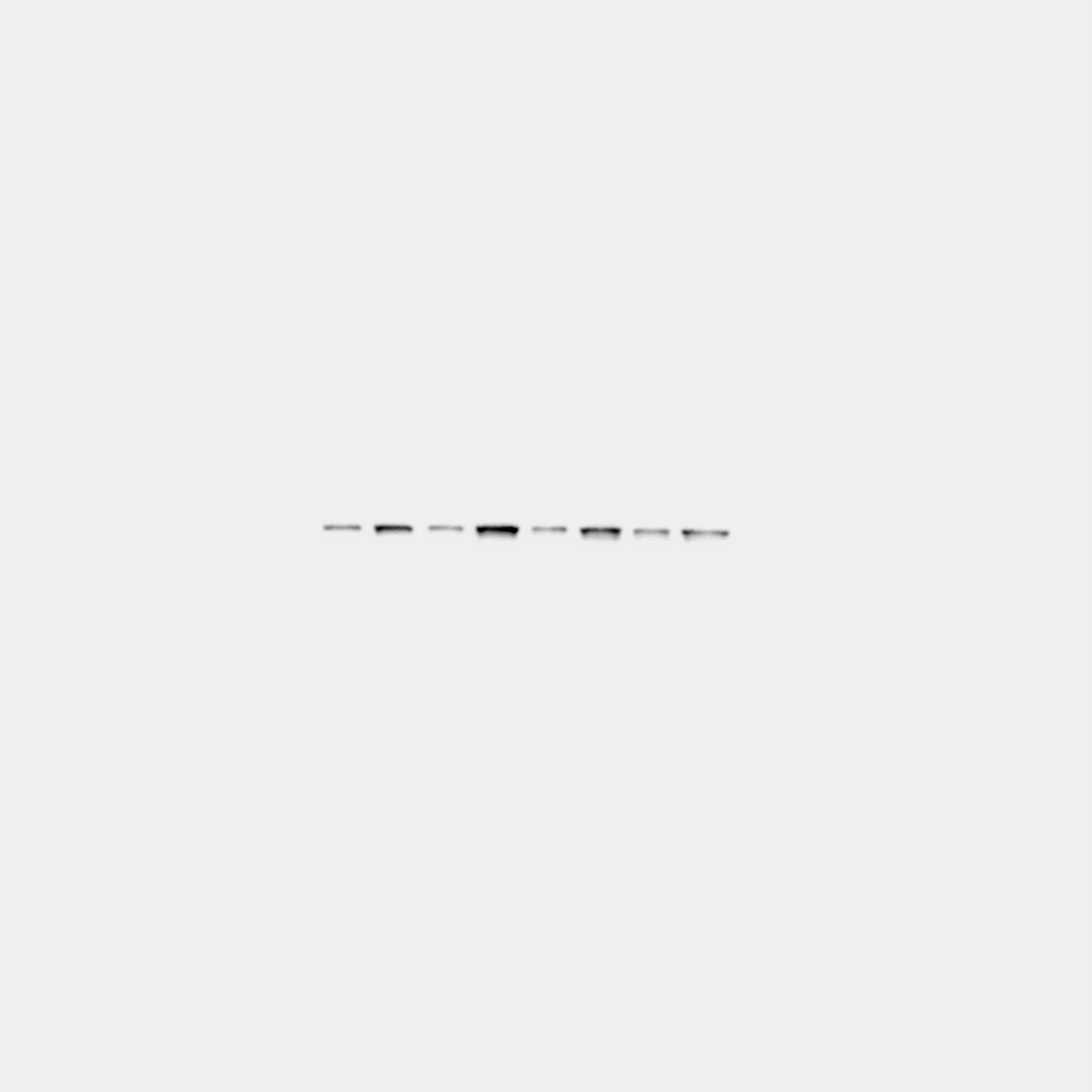


**SOX2**

Figure 6D


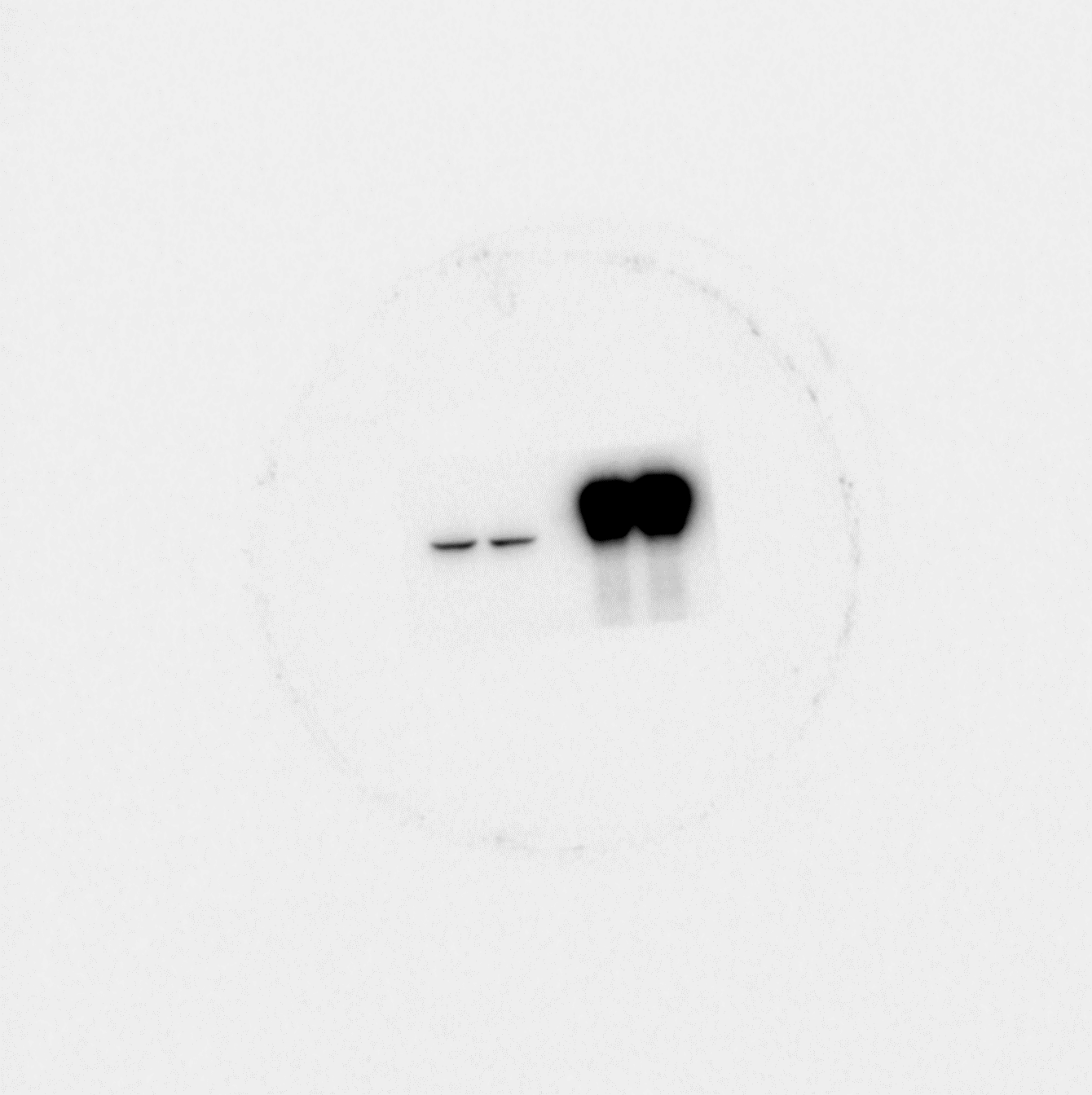

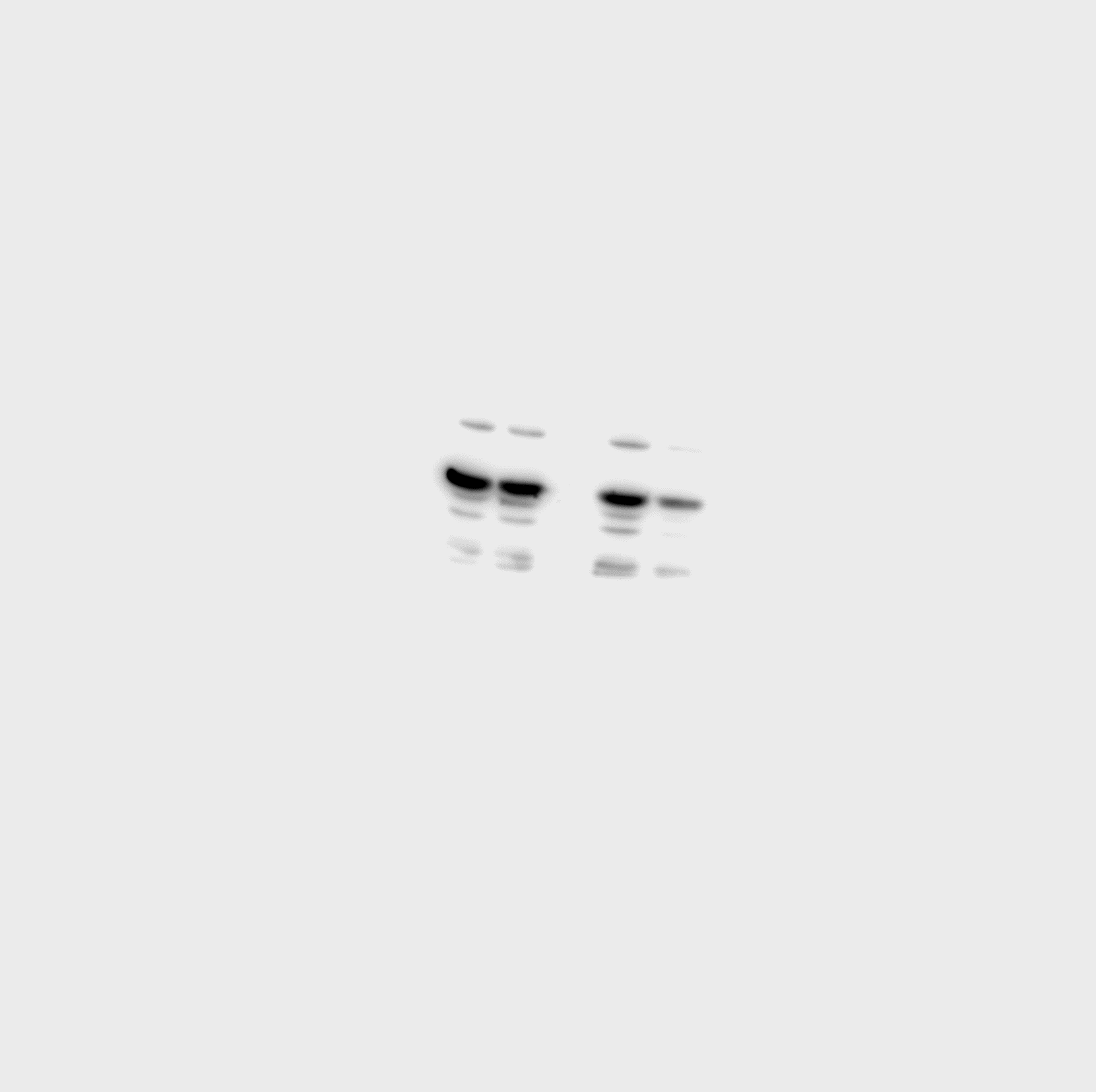


**YBX1**

**ILF2**

Figure 7J


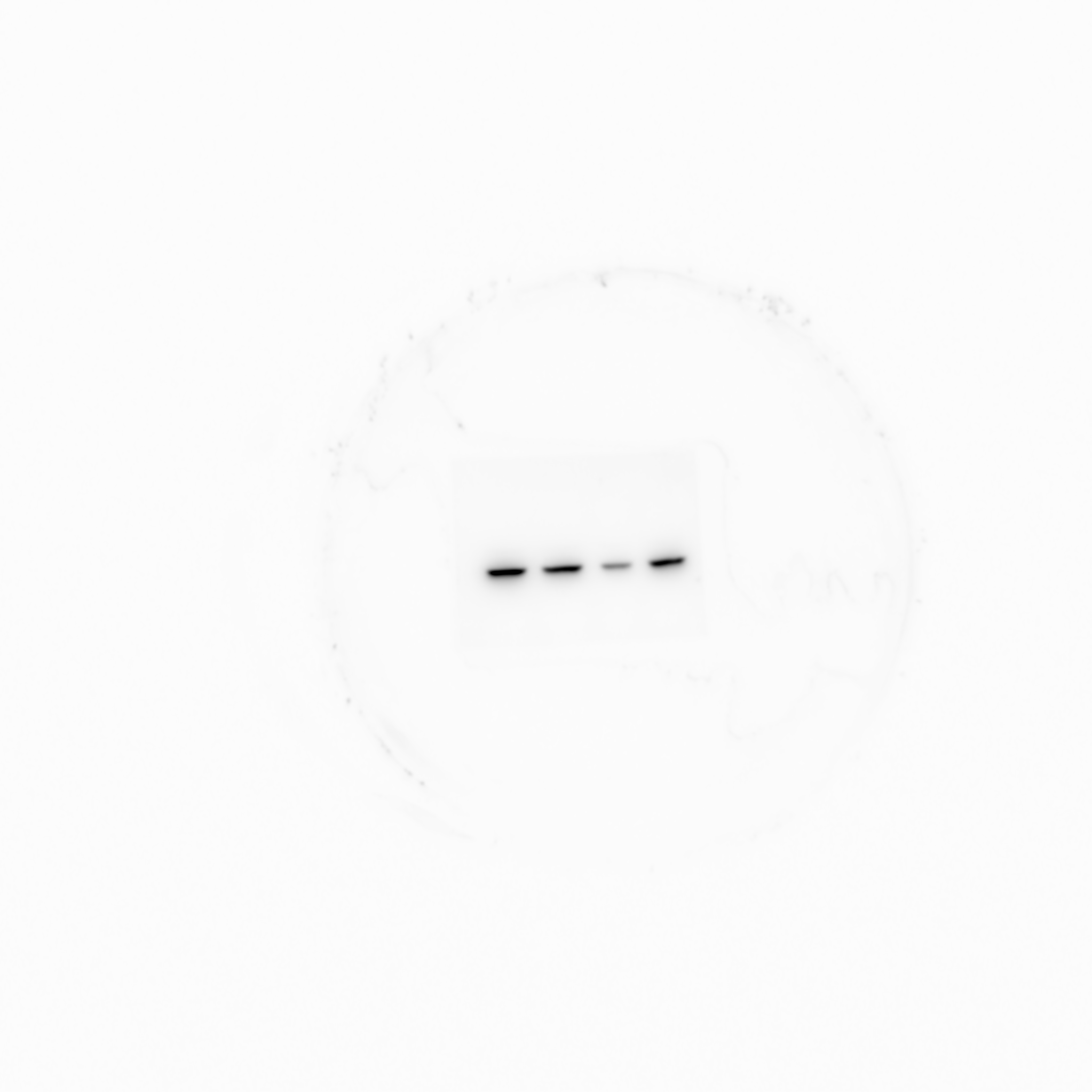

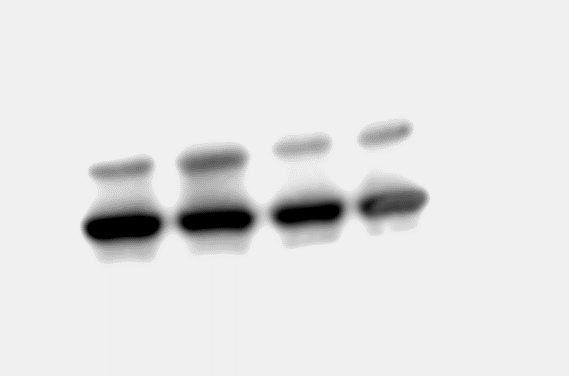

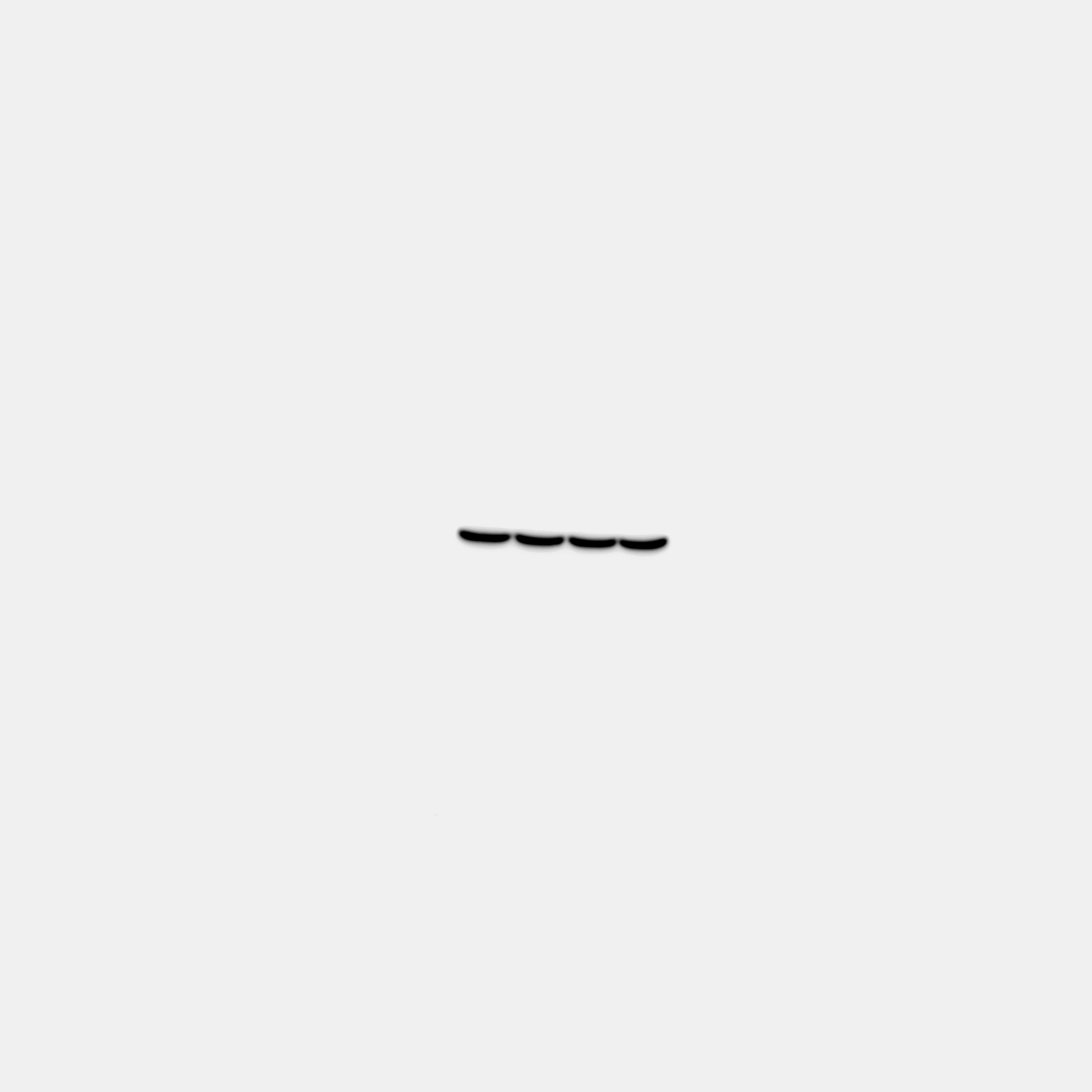

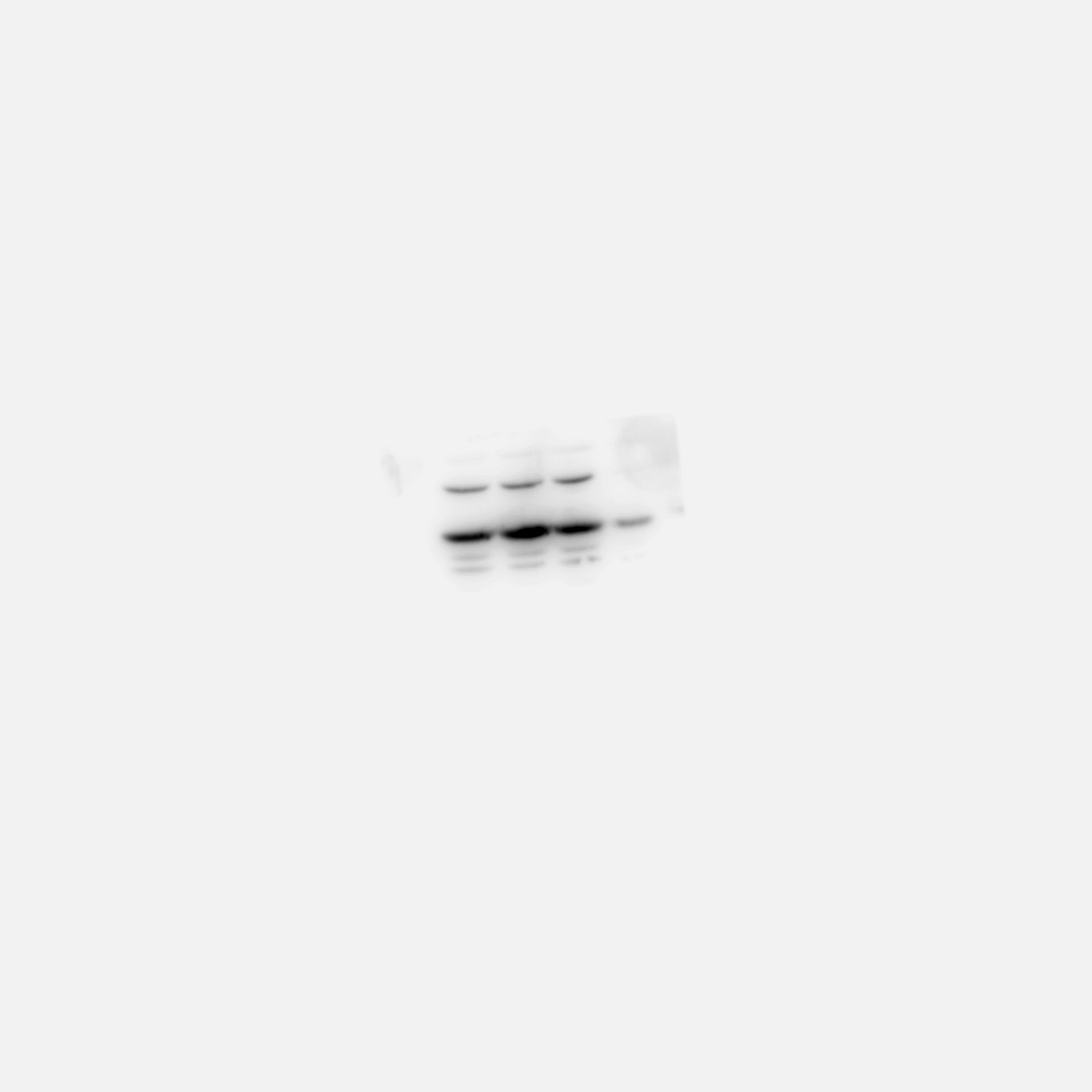


**β-Actin**

**SOX2**

**ILF2**

**YBX1**
